# MicroRNA-21 as a diagnostic and prognostic biomarker of lung cancer: a systematic review and meta-analysis

**DOI:** 10.1042/BSR20211653

**Published:** 2022-05-10

**Authors:** Wei Wang, Xinyao Li, Chengfei Liu, Xin Zhang, Ying Wu, Mingxin Diao, Siyu Tan, Shubin Huang, Yin Cheng, Tao You

**Affiliations:** 1 Department of Cardiac Surgery, The First Hospital of China Medical University, Shenyang, Liaoning, China; 2 The First Clinical Medical College of Lanzhou University, Lanzhou, Gansu, China; 3 Gansu University of Chinese Medicine, Lanzhou, Gansu, China; 4 The First Clinical Medical College of Anhui Medical University, Hefei, Anhui, China; 5 Department of Cardiovascular Surgery, Gansu Provincial Hospital, Lanzhou, Gansu, China

**Keywords:** Diagnosis, meta analysis, MicroRNA-21, Prognosis

## Abstract

**Background:** The relationship between microRNA-21 (miRNA-21) and pathogenesis of lung cancer is a considerable focus of research interest. However, to our knowledge, no in-depth meta-analyses based on existing evidence to ascertain the value of miRNA-21 in diagnosis and clinical prognosis of lung cancer have been documented.

**Methods:** We comprehensively searched all the literature pertaining to ‘miRNA-21’ and ‘lung cancer’ from four databases from the period of inception of each database until May 2020. Using specific inclusion and exclusion criteria, the literature for inclusion was identified and the necessary data extracted.

**Results:** In total, 46 articles were included in the meta-analysis, among which 31 focused on diagnostic value and 15 on prognostic value. Combined sensitivity (SEN) of miRNA-21 in diagnosis of lung cancer was 0.77 (95% confidence interval (CI): 0.72–0.81), specificity (SPE) was 0.86 (95% CI: 0.80–0.90), diagnostic odds ratio (DOR) was (95% CI: 12–33), and area under the SROC curve (AUC) was 0.87 (95% CI: 0.84–0.90). No significant correlations were observed between abnormal expression of miRNA-21 and gender, smoking habits, pathological type and clinical stage of lung cancer (*P*>0.05). In terms of overall survival (OS), univariate analysis (hazards ratio (HR) = 1.49, 95% CI: 1.22–1.82) revealed high expression of miRNA-21 as an influencing factor for lung cancer. MiRNA-21 was confirmed as an independent risk factor for poor prognosis in multivariate analysis (HR = 1.65, 95% CI: 1.24–2.19).

**Conclusion:** MiRNA-21 has potential clinical value in the diagnosis and prognosis of lung cancer and may serve as an effective diagnostic marker and therapeutic target in the future.

## Introduction

Lung cancer is a malignant tumor with the highest incidence among both men and women worldwide [1]. According to the ‘Annual Report to the Nation on the Status of Cancer’, lung cancer is the predominant cause of tumor-related mortality, with a 5-year survival rate of ∼15–20% [2,3]. In terms of pathological type, ∼15% cases are small-cell lung cancer (SCLC) and 85% represent non-small cell lung cancer (NSCLC). Existing data show that the 5-year overall survival (OS) rate of patients with early lung cancer (stage I) is ∼80%, while the rates for locally advanced or metastatic lung cancer (stage III or IV) are 37 and 6%, respectively [4], clearly indicating that poor diagnosis and high progression rates contribute to low OS. The main existing methods of diagnosis include chest X-ray, low-dose spiral CT (LDCT), sputum cytology and percutaneous biopsy. Although specimens obtained via puncture biopsy are considered the gold standard for diagnosis, they are prone to complications, such as pneumothorax and bleeding. Moreover, the examination is invasive, specimens are hard to obtain and there is a high risk of false results [5]. However, the sensitivity (SEN) and specificity (SPE) of other techniques are unable to meet the requirements of early diagnosis [6]. Identification of non-invasive markers for lung cancer with high SEN and SPE thus remains a significant clinical challenge. In the third edition of the ‘National Comprehensive Cancer Network’ (NCCN) clinical practice guidelines (2018), several risk factors of lung cancer have been highlighted. However, there is a significant void in the dimension of analysis and evaluation of prognosis [7]. MicroRNAs are clearly involved in cancer-related cell growth and tissue differentiation and have been shown to serve as negative regulators. Their abnormal expression and mutation are significantly associated with the occurrence of human cancer [8] and stable microRNAs in plasma may therefore have utility as potential biomarkers for lung cancer diagnosis and prognosis.

MicroRNAs (miRNAs) are a class of natural non-coding small RNA molecules ∼21–25 nucleotides in length, which influence the stability of mRNA and translation processes by binding to incomplete complementary sites in the 3′ untranslated region (UTR) of their mRNA targets to participate in gene expression after transcription [8] and consequently play critical regulatory roles [9]. In the early stages of development of several cancer types, miRNA imbalance is detected in peripheral blood and significant correlations observed among its expression, degree of change and prognosis of cancer [10]. MicroRNA-21 (miRNA-21) is one of the earliest identified and most extensively investigated miRNAs [11]. The gene encoding miRNA-21 is located at 17q23.2. As a tumor-promoting miRNA, miRNA-21 indirectly stimulates cancer occurrence and development through regulation of multiple downstream tumor suppressor genes [12]. MiRNA-21 is expressed abnormally in multiple cancer cell types and increased in most malignant tumors including glioma, lung cancer and breast cancer. An earlier study reported markedly elevated expression of miRNA-21 in peripheral blood of patients with lung cancer, suggesting an association with disease occurrence [13]. The significant correlation of miRNA-21 with lung cancer development supports its potential as a biomarker for early diagnosis of the disease [14–16]. Accumulating studies have focused on the utility of miRNA-21 as a predictor of poor prognosis [17–23]. MiRNA-21 specifically down-regulates the tumor suppressor genes, *PTEN* and *TPM1*, and may thus play a carcinogenic role [24,25].

While studies to date clearly support the potential utility of miRNA-21 as a biomarker for early diagnosis and poor prognosis of patients with lung cancer, limitations include small sample sizes of each independent study and inconsistency of individual research conclusions. Accordingly, a meta-analysis of the diagnostic and prognostic value of miRNA-21 in lung cancer was conducted in the present study, with a view to provide a platform for early diagnosis, risk and prognosis assessment.

## Materials and methods

The search terms were determined using the principle of ‘PICO’ and all publications from the inception of the database up to May 2020 searched from four databases (PubMed, Web of Science, CNKI, Wanfang). Simultaneously, a search was conducted on references of the included literature to comprehensively identify all related materials on ‘microRNA-21’ and ‘Lung Cancer’. The search terms included ‘microRNA-21’, ‘miRNA-21’, ‘miR-21’, and ‘Lung Cancer’. Taking PubMed as an example, the search strategy is presented in Table 1.

**Table 1:** Taking Pubmed as an example, the retrieval process was shown in the following table

Set	Query
#1	miRNA-21 [Title/Abstract]
#2	microRNA-21 [Title/Abstract]
#3	miR-21 [Title/Abstract]
#4	hsa-miR-21 [Title/Abstract]
#5	#1 OR #2 OR #3 OR #4
#6	Lung Neoplasms [MeSH]
#7	Carcinoma, Non-Small-Cell Lung [MeSH]
#8	Carcinoma, Small Cell [MeSH]
#9	Cancer of Lung [Title/Abstract]
#10	Lung Cancer [Title/Abstract]
#11	Pulmonary Cancer [Title/Abstract]
#12	Pulmonary Neoplasms [Title/Abstract]
#13	Cancer of the Lung [Title/Abstract]
#14	Neoplasms, Lung [Title/Abstract]
#15	Neoplasms, Pulmonary [Title/Abstract]
#16	Cancer, Lung [Title/Abstract]
#17	Cancer, Pulmonary [Title/Abstract]
#18	Cancers, Lung [Title/Abstract]
#19	Cancers, Pulmonary [Title/Abstract]
#20	Lung Cancers [Title/Abstract]
#21	Lung Neoplasm [Title/Abstract]
#22	Neoplasm, Lung [Title/Abstract]
#23	Neoplasm, Pulmonary [Title/Abstract]
#24	Pulmonary Cancers [Title/Abstract]
#25	Pulmonary Neoplasm [Title/Abstract]
#26	Carcinoma, Non-Small Cell Lung [Title/Abstract]
#27	Non-Small Cell Lung Cancer [Title/Abstract]
#28	Non-Small-Cell Lung Carcinoma [Title/Abstract]
#29	Nonsmall Cell Lung Cancer [Title/Abstract]
#30	Carcinoma, Non Small Cell Lung [Title/Abstract]
#31	Carcinomas, Non-Small-Cell Lung [Title/Abstract]
#32	Lung Carcinoma, Non-Small-Cell [Title/Abstract]
#33	lung Carcinomas, Non-Small-Cell [Title/Abstract]
#34	Non Small Cell Lung Carcinoma [Title/Abstract]
#35	Non-Small-Cell Lung Carcinomas [Title/Abstract]
#36	Oat Cell Carcinoma of Lung [Title/Abstract]
#37	Carcinoma, Small Cell Lung [Title/Abstract]
#38	Oat Cell Lung Cancer [Title/Abstract]
#39	Small Cell Cancer Of The Lung [Title/Abstract]
#40	Small Cell Lung Cancer [Title/Abstract]
#41	Small Cell Lung Carcinoma [Title/Abstract]
#42	#6 OR #7 OR #8 OR #9 OR #10 OR #11 OR #12 OR #13 OR #14 OR #15 OR #16 OR #17 OR #18 OR #19 OR #20 OR #21 OR #22 OR #23 OR #24 OR #25 OR #26 OR #27 OR #28 OR #29 OR #30 OR #31 OR #32 OR #33 OR #34 OR #35 OR #36 OR #37 OR #38 OR #39 OR #40 OR #41
#43	survival [Title/Abstract]
#44	prognosis* [Title/Abstract]
#45	#43 OR #44
#46	diagnosis* [Title/Abstract]
#47	#45 OR #46
#48	#5 AND #42 AND #47

### Literature inclusion and exclusion criteria

Inclusion criteria were as follows: (1) miRNA-21 studies on diagnosis and prognosis of lung cancer published at home and abroad, (2) diagnosis of lung cancer based on miRNA-21 supported by pathological evidence, (3) lung cancer patients as the study group and healthy individuals or patients with benign lung diseases as the control group, (4) availability of survival data in prognosis studies, such as OS, relapse-free survival (RFS), progression-free survival (PFS) or disease-free survival (DFS), with survival results obtained either directly or indirectly.

Exclusion criteria were as follows: (1) non-English or Chinese literature, (2) reviews, conference summaries and animal experiments, (3) repeat publications or insufficient sample size for analysis, (4) combined analysis of miRNA-21 with other genes in diagnosis of lung cancer or lack of available data.

### Data extraction and quality assessment

After identifying the literature for inclusion, two researchers screened and extracted the relevant data. For each study, we extracted the first author’s name, publication year, country, population characteristics (such as sample size, race, specimen type, lung cancer stage), and other basic information. For diagnostic studies, area under the SROC curve (AUC), SEN, SPE and other key data were obtained and a 2 × 2 contingency table designed including false negative (FN), true negative (TN), true positive (TP) and false positive (FP) results. In prognostic analyses, in addition to basic information, we collated data on high/low expression of miRNA-21 in relation to gender, smoking, lung cancer classification and staging and extracted Hazards Ratio (HR), Odds Ratio (OR), 95% Confidence Interval (95% CI) and other relevant measures of association. After completion of data extraction, results were summarized, sorted through Microsoft Excel 2019 spreadsheets and tabulated for ease of analysis.

The quality of all included diagnostic studies was evaluated with the ‘Quality Assessment of Diagnostic Accuracy Studies-2’ *(QUADAS-2)* [26]. After detailed examination of the full text, four items (Case selection, Test to be evaluated, Gold standard, Case flow and progress) were analyzed according to the three judgment criteria of ‘Yes’, ‘No’ and ‘Unclear’. Prognostic studies were assessed according to ‘Newcastle–Ottawa Scale’ (NOS) criteria based on three quality parameters (selectivity, comparability and exposure factors) [27,28]. The quality of studies was considered high at scores ≥ 7.

The studies were cross-checked, reviewed independently by two researchers and discussed in case of dispute until a consensus was reached.

### Statistical analysis

STATA 16.0 and Review Manager 5.3 were applied for statistical analysis of all studies. For diagnostic analyses, the classification variable model was used to calculate the combined results of SEN, SPE, positive likelihood ratio (PLR), negative likelihood ratio (NLR), diagnostic odds ratio (DOR) and 95% CI, and ROC curves were generated. In the meta-analysis of prognostic studies, HR and 95% CI were combined to evaluate the effects of high expression of miRNA-21 on survival times of lung cancer patients. According to the study of Greenland et al. [29], the differences between OR, relative risk (RR) and hazard ratio (HR) may be negligible and therefore OR and RR values obtained from the included studies could be regarded as HR. Q-value and *I^2^* tests were applied to evaluate heterogeneity among studies. At *P*<0.05 and *I^2^* > 50%, significant heterogeneity between the studies was present and a random-effects model was used for data consolidation, while at *P*>0.05, *I^2^* < 50%, the fixed-effects model was used. Publication bias was assessed using Deek’s Funnel plot and the Begg’s/Egger’s tests. Data were considered statistically significant at *P*<0.05. In case of identified univariate variables that influenced heterogeneity between included studies, subgroup analysis was conducted. For studies showing significant heterogeneity or high risk of bias, sensitivity analysis was conducted to validate the stability of the combined effect.

## Results

### Literature retrieval and research features

**Figure 1:**
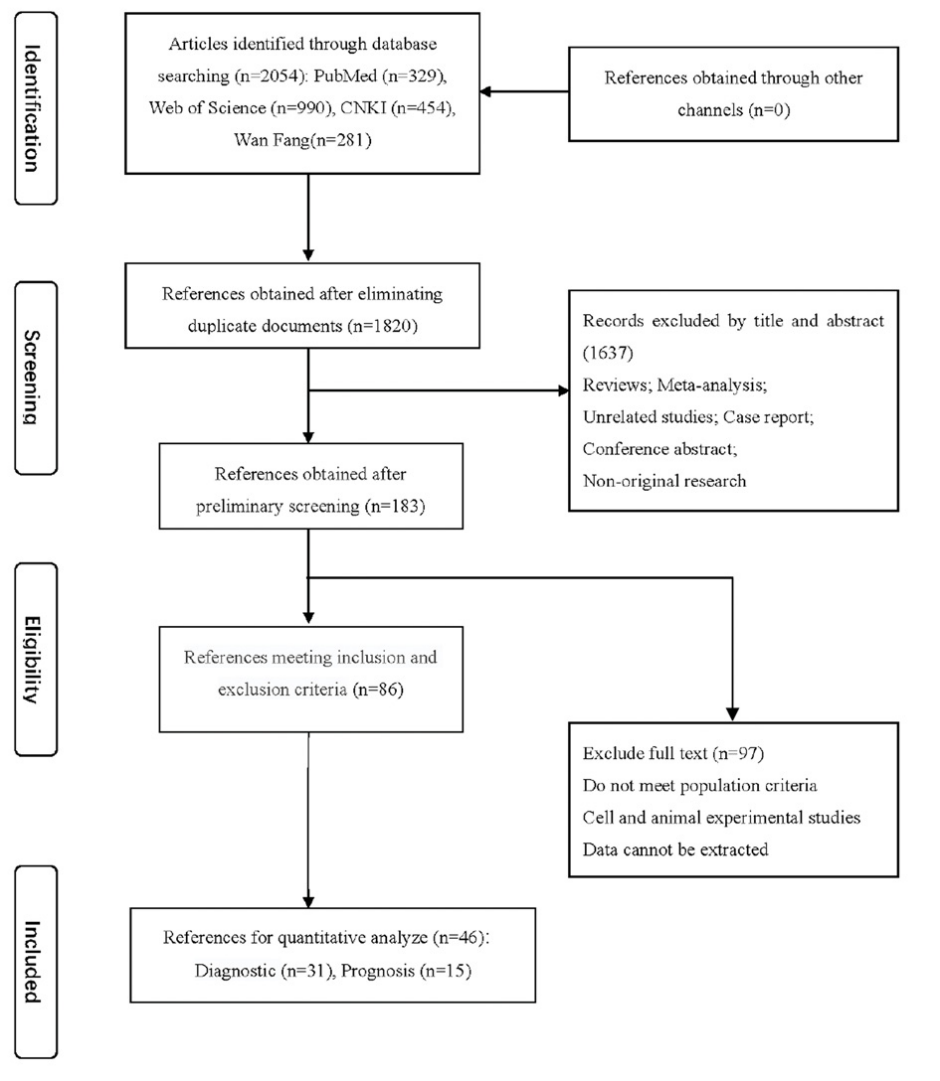
Flow chart of the literature search for the meta-analysis

A total of 2054 related studies were retrieved according to the established search terms. Following elimination of repeat studies, 183 were preliminarily screened. Based on the inclusion and exclusion criteria, 86 articles were selected. Finally, 46 articles [14,15,22,30–72] were included in the meta-analysis based on the terms ‘Lung cancer’ and ‘microRNA-21’. Among these, 31 [14,15,30–58] reported the diagnostic value of miRNA-21 and 15 [22,69–72] focused on its prognostic value. The search flow chart is shown in Figure 1.

### Meta-analysis of studies on the diagnostic value of miR-21

A total of 3707 patients were included in the diagnostic meta-analysis (2124 patients in the lung cancer group (non-small cell and small-cell lung cancer) and 1583 patients in the control group (healthy subjects and patients with benign nodules)). Overall, we retrieved 28 articles [14,15,30–37,40–44,46–58] on NSCLC from the literature (one on lung adenocarcinoma). In addition, related studies by Ke et al. [30] on the expression of miRNA-21 and clinical stages I and III of NSCLC were divided into two baseline groups (Table 2).

**Table 2:** Characteristics of diagnostic clinical trials included in the meta-analysis and the diagnostic efficacy of miRNA-21 for lung cancer

Author	Year	Country	Cancer Type	Specimen	Case/Control	TP	FP	FN	TN	AUC (95% CI)	SEN	SPE
Qing [14]	2014	China	NSCLC	Plasma	126/60	84	19	42	41	0.77	0.67	0.68
Hui [15]	2016	China	NSCLC	Plasma	129/83	100	12	29	71	0.838	0.775	0.855
Yang (I) [30]	2019	China	NSCLC	Serum	89/90	80	1	9	89	0.966	0.8989	0.9889
Yang (III) [30]	2019	China	NSCLC	Serum	89/90	87	1	2	89	0.971	0.973	0.993
Jing [31]	2016	China	NSCLC	Serum	68/64	63	25	5	39	0.873	0.932	0.607
Qixin [32]	2018	U.S.A.	NSCLC	Plasma	40/29	30	11	10	18	0.92	0.7576	0.6364
Abu-Duhier [33]	2018	Saudi Arabia	NSCLC	Plasma	80/80	64	16	16	64	0.8913	0.8	0.8
Abdollahi [34]	2019	Iran	NSCLC	Serum	43/43	39	14	4	29	0.85	0.9	0.67
Bole [35]	2012	China	NSCLC	Serum	82/50	39	6	43	44	0.696	0.478	0.88
Yanzhao [36]	2011	China	NSCLC	Serum	20/10	16	0	4	10	0.921 ± 0.045	0.788	1
Dongfang [37]	2013	China	NSCLC	Plasma	34/32	18	9	16	23	0.71492	0.529	0.719
Bing [38]	2012	China	LC	Serum	31/39	27	10	4	29	0.88	0.871	0.744
Sheng [39]	2018	China	LC	Serum	50/24	30	8	20	16	0.653	0.6	0.667
Xiaoqin [40]	2018	China	NSCLC	Serum	167/128	108	46	59	82	0.831	0.647	0.641
Juan [41]	2011	China	NSCLC	Plasma	77/36	47	6	30	30	0.729	0.6104	0.8333
Ying [42]	2010	U.S.A.	NSCLC	Sputum	23/17	16	0	7	17	0.902 ± 0.054	0.6966	1
Dapeng [43]	2019	China	NSCLC	Serum	85/30	51	0	34	30	0.854	0.6	1
Hailei [44]	2019	China	NSCLC	Serum	95/30	80	3	15	27	/	0.8421	0.9
Junting [45]	2014	China	LC	Serum	90/130	64	23	26	107	0.808	0.711	0.823
Yuqiao [46]	2017	China	NSCLC	Serum	50/60	38	12	12	48	0.882	0.75	0.8
Shirong [47]	2019	China	NSCLC	Serum	32/20	27	0	5	20	0.966	0.844	1
Xi [48]	2017	China	NSCLC	Tissue	32/20	21	0	11	20	0.85	0.65	1
Xin [49]	2017	China	NSCLC	Plasma	56/47	43	11	13	36	0.825	0.768	0.766
Jiajia [50]	2012	China	NSCLC	Tissue	31/10	23	2	8	8	0.785	0.733	0.8
Juan [51]	2016	China	NSCLC	Serum	64/30	48	9	15	21	0.775	0.7619	0.7
Xiaoqian [52]	2013	China	NSCLC	Sputum	24/11	20	3	4	8	0.863	0.833	0.75
Zhennan [53]	2015	China	NSCLC	Tissue	150/150	132	45	18	105	0.8665	0.8824	0.6997
Yongpan [54]	2017	China	NSCLC-AC	Plasma	28/28	23	1	5	27	0.88	0.821	0.964
Abd-El-Fattah [55]	2013	Egypt	NSCLC	Serum	65/37	56	5	9	32	0.47	0.857	0.865
Jun [56]	2011	U.S.A.	NSCLC	Plasma	58/29	39	2	19	27	0.816	0.675	0.941
Mozzoni [57]	2013	Italy	NSCLC	Plasma	54/46	27	4	27	42	0.74	0.5	0.923
Juan [58]	2011	China	NSCLC	Serum	63/30	48	9	15	21	0.775	0.762	0.7

Abbreviations: LC, lung cancer; NSCLC-AC, lung adenocarcinoma.

#### Combined results

**Figure 2:**
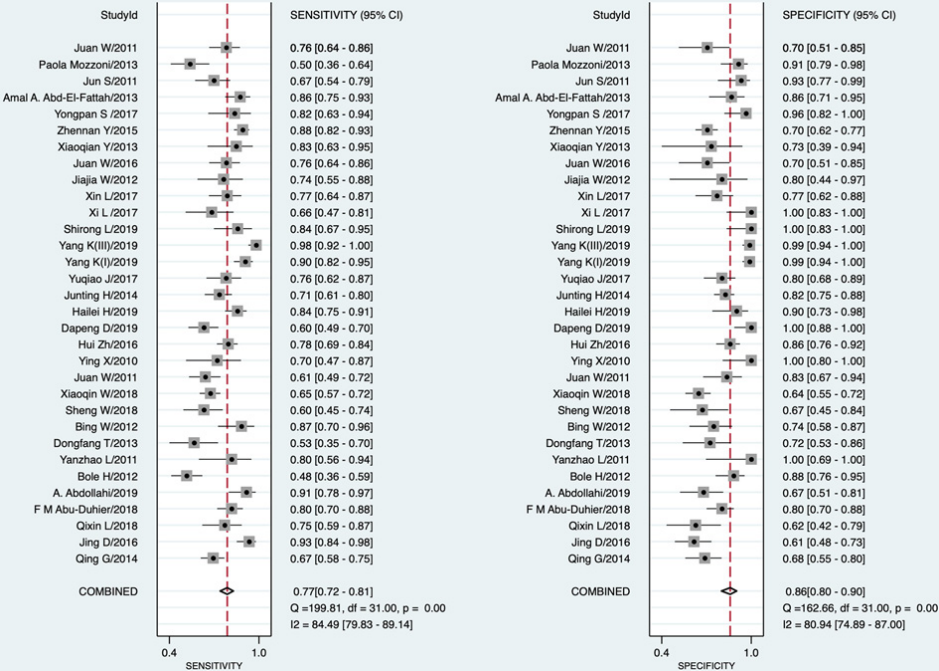
Forest plot of sensitivities and specificities of miRNA-21 in diagnosis of lung cancer

**Figure 3:**
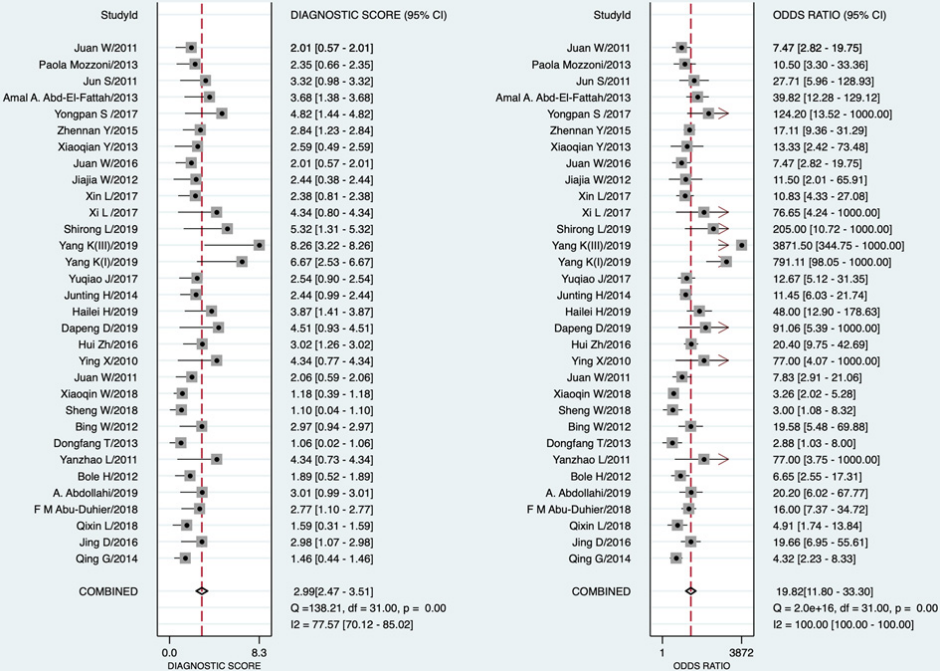
Forest plot of OR of miRNA-21 in diagnosis of lung cancer

**Figure 4:**
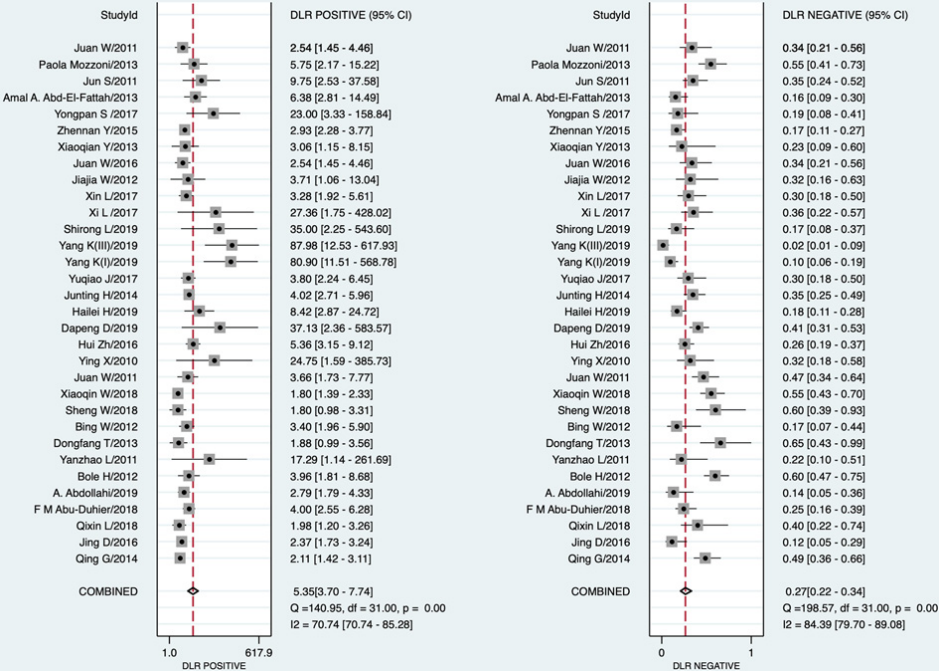
Forest plots of DLR positivity and DLR negativity of miRNA-21 in diagnosis of lung cancer

**Figure 5:**
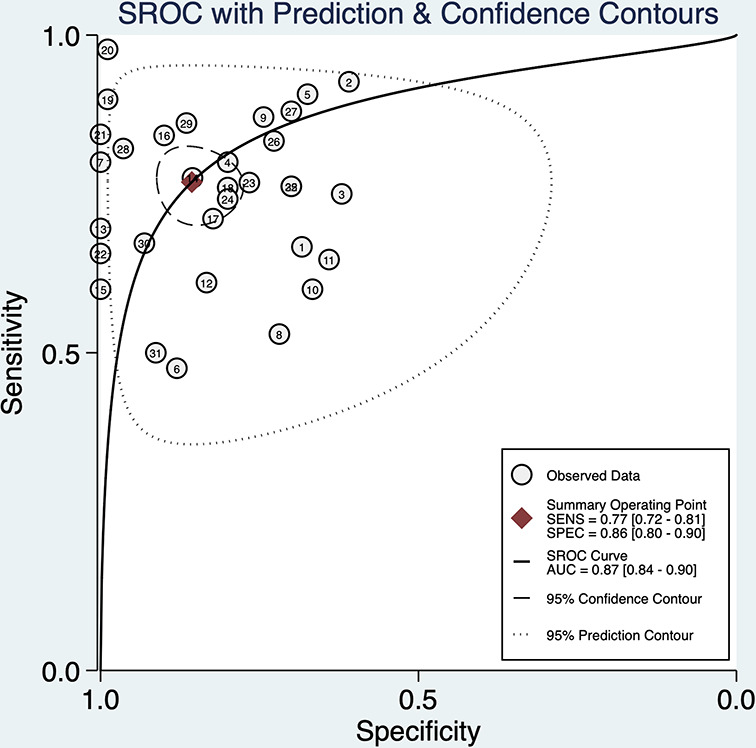
SROC curves of miRNA-21 for diagnosis of lung cancer

**Figure 6:**
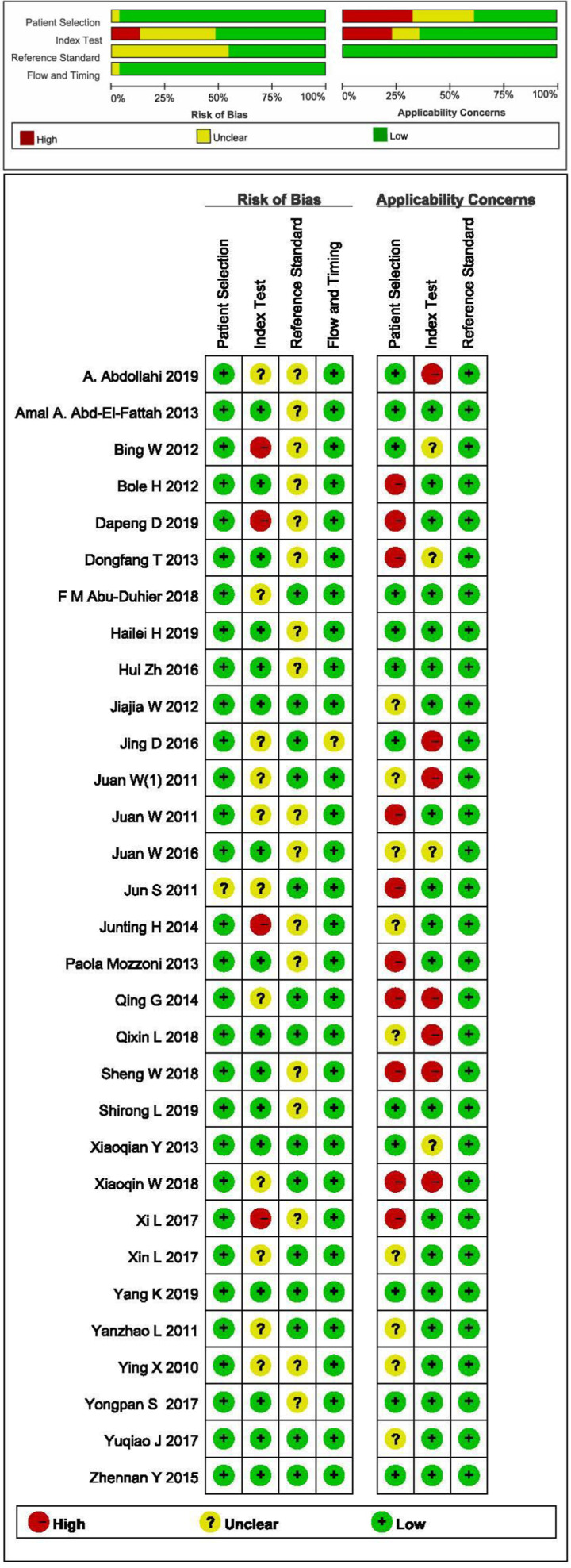
Quality assessment of diagnostic accuracy for the included studies

STATA 16.0 analysis of 32 studies [14,15,30–58] showed combined sensitivity of miRNA-21 for diagnosis of lung cancer of 0.77 (95% CI: 0.72–0.81), specificity of 0.86 (95% CI: 0.80–0.90), PLR of 5.4 (95% CI: 3.7–7.7), NLR of 0.27 (95% CI: 0.22–0.34), DOR of 20 (95% CI: 12–33) and AUC of 0.87 (95% CI: 0.84–0.90). The results are presented in Figures 2-5. Evaluation of the included studies using a QUADAS-2 rating scale revealed high overall quality (Figure 6).

#### Subgroup analysis

To further explore the diagnostic performance of miRNA-21, we performed subgroup analysis according to race, specimen and lung cancer type. The ethnic groups examined were Asian (*n*=24) [14,15,30,31,35–41], North American (*n*=3) [32,42,56], Middle Eastern (*n*=2) [33,34], European (*n*=1) [57] and African (*n*=1) [55]. Specimens included serum, plasma and others (such as biopsy tissue/sputum). Due to insufficient data on SCLC patients, only results from NSCLC patients were combined for lung cancer types. Specific merged results are shown in Table 5 and Supplementary Figures S1–S24.

#### Sensitivity analysis

Goodness-of-fit and bivariate normality results supported the suitability of the bivariate random-effects model. Influence analysis revealed that studies of Jing et al. [31], Dapeng et al. [44] and two reports by Yang et al. [30] accounted for a higher proportion of the total weight of all studies (see Figure 8). Outlier detection analysis indicated that the two studies by Yang et al. [30] could be the cause of heterogeneity. After excluding the abnormal data [30], the sensitivity value changed from 0.77 to 0.75, specificity from 0.86 to 0.82, and AUC from 0.87 to 0.85.

#### Publication bias

Deek’s funnel plot was used to detect publication bias. The results indicated no significant publication bias and good article consistency (t = 0.15, *P*>0.88).

### Meta-analysis of prognostic factors

**Figure 7:**
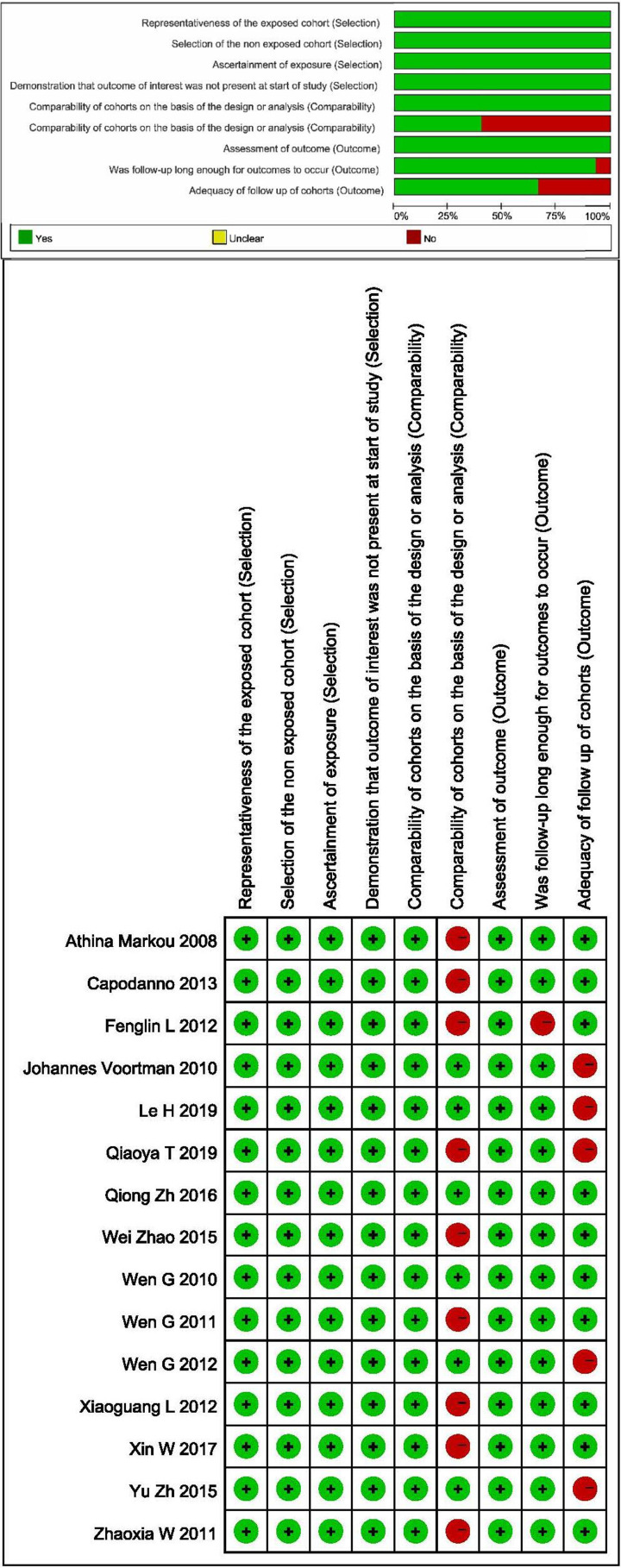
Quality assessment of prognostic accuracy for the included studies

A total of 1603 participants from 15 studies [22,50–72] were included in our meta-analysis of prognosis. Among these studies, 12 provided data on OS of lung cancer patients [60,62–72], 1 on PFS [61], 1 on DFS [22]. A study by Capodanno et al. [59] simultaneously investigated OS and PFS of lung cancer patients. Regarding type of lung cancer, 11 studies were on NSCLC [22,59–63,67–70,72] and 1 on squamous cell lung carcinoma [71]. In terms of race, 12 studies investigated Chinese patients [22,60–67,70–72] and the other 3 were on Italian [59], Greek [69] and French [68] patients. The group of Xiaoguang [60] analyzed expression of miRNA-21 in both tissue and serum, four studies reported miRNA-21 expression in serum [61,64,67,72] and ten focused on miRNA-21 in tissue [22,59,62,63,75,66,68–71] (Tables 3 and 4). The quality of the included studies was evaluated via NOS. The scores ranged from 7 to 9, signifying good quality of all the included studies. The results are presented in Figure 7.

**Figure 8:**
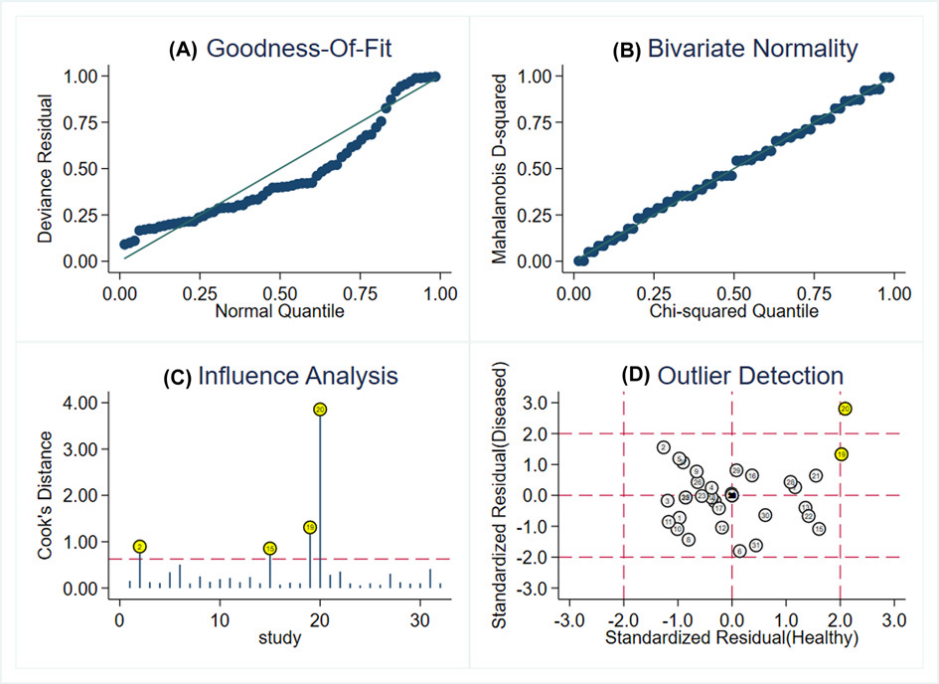
Influence analysis and outlier detection (**A**) Goodness-of-fit, (**B**) bivariate normality, (**C**) influence analysis, and (**D**) outlier detection. SROC curves of miRNA-21 for diagnosis of lung cancer.

**Table 3:** Baseline characteristics of correlation studies

Author	Year	Country	Cancer type	Total number	Speci- men	Gender				Smoking History				Histological type of NSCLC				Pathological staging							
						Male		Female		Yes		No		AD		SC		1		2+		1-2		3+	
						High	Low	High	Low	High	Low	High	Low	High	Low	High	Low	High	Low	High	Low	High	Low	High	Low
Wen [22]	2012	China	NSCLC	58	Tissue	14	18	15	11	/	/	/	/	17	16	11	13	7	9	22	19	/	/	/	/
Capodanno [59]	2013	Italy	NSCLC	80	Tissue	28	27	12	13	6	7	11	17	31	24	7	14	7	5	24	16	/	/	/	/
Yu [62]	2015	China	NSCLC	32	Tissue	15	4	7	6	/	/	/	/	11	4	11	6	/	/	/	/	5	6	17	4
Fenglin [65]	2012	China	NSCLC	48	Tissue	14	16	7	11	/	/	/	/	9	9	10	13	/	/	/	/	/	/	/	/
Zhaoxia [67]	2011	China	NSCLC	88	Serum	25	21	24	18	27	23	22	16	20	17	11	10	/	/	/	/	21	26	28	31
Markou [69]	2008	Athens	NSCLC	48	Tissue	/	/	/	/	23	21	2	2	14	11	11	12	/	/	/	/	17	15	8	8
Wen [71]	2011	China	SCC	30	Tissue	/	/	/	/	4	2	11	13	/	/	/	/	8	9	7	6	/	/	/	/

Abbreviations: AC, adenocarcinoma of the lung cancer; SC, lung squamous cancer; SCC, small cell lung cancer; 1, lung cancer stage 1; 2+, lung cancer stages 2–4; 1–2, lung cancer stages 1–2; 3+, lung cancer stages 3–4.

**Table 4:** Characteristics and quality assessment of prognostic clinical trials included in the meta-analysis

Author	Year	Country	Cancer type	Total number	Specimen	Results	Follow-up (month)	Hazard Ratio (95%CI), P		NOS
								Univariate	Multivariate	
Wen [22]	2012	China	NSCLC	58	Tissue	DFS	13–23	3.265 (1.276–8.357), *P*=0.014	2.820 (1.091–7.285), *P*=0.032	8
Capodanno [59]	2013	Italy	NSCLC	80	Tissue	OS	7–98	2.55 (0.62–10.56), *P*=0.0045	/	8
					Tissue	PFS	7–98	1.17 (0.43–3.2), *P*=0.0003	/	
Xiaoguang [60]	2012	China	NSCLC	70	Tissue	OS	24	3.187 (0.368–7.592), *P*=0.123	/	8
					Serum	OS	24	4.316 (1.265–19.206), *P*=0.046	/	
Qiong [61]	2016	China	NSCLC	51	Serum	PFS	24	/	1.619 (1.369–3.221), *P*=0.030	9
Yu [62]	2015	China	NSCLC	32	Tissue	OS	6–31	1.94 (0.51–7.32)	/	8
Xin [63]	2017	China	NSCLC	152	Tissue	OS	72–120	1.127 (1.037–1.226), *P*=0.005	1.149 (1.055–1.250), *P*=0.001	8
Qiaoya [64]	2019	China	LC	87	Serum	OS	/	1.68 (0.85–3.29)	/	7
Fenglin [65]	2012	China	LC	48	Tissue	OS	60	2.05 (1.05–3.97), *P*=0.027	/	7
Le [66]	2019	China	LC	85	Tissue	OS	60	1.12 (0.49–2.58)	/	8
Zhaoxia [67]	2011	China	NSCLC	88	Serum	OS	1–73	2.01 (1.49–2.72), *P*=0.018	2.01 (1.78–3.26), *P*=0.015	8
Voortman [68]	2010	France	NSCLC	639	Tissue	OS	/	0.81 (0.65–1.01), *P*=0.06	/	8
Markou [69]	2008	Athens	NSCLC	48	Tissue	OS	39	1.90 (0.74–4.88), *P*=0.027	2.533 (1.066–6.020), *P*=0.035	8
Wen [70]	2010	China	NSCLC	56	Tissue	OS	42–63	2.710 (1.392–5.275), *P*=0.003	5.993 (2.518–14.264), *P*=0.000	9
Wen [71]	2011	China	SCC	30	Tissue	OS	48–60	1.246 (1.093–1.419), *P*=0.001	1.293 (1.123–1.489), *P*=0.000	8
Zhao [72]	2015	China	NSCLC	80	serum	OS	12–48	2.18 (1.28–3.7), *P*=0.001	/	8

Abbreviation: LC, lung cancer.

**Table 5:** Summary results for diagnostic accuracy of miRNA-21 for lung cancer

Analysis	*n*	SEN (95% CI)	*I^2^*	SPE (95% CI)	*I^2^*	PLR (95% CI)	NLR (95% CI)	DOR (95% CI)	AUC (95% CI)	Deeks' Funnel Plot	
										*t*	P (t)
Ethnicity											
China	25	0.77 (0.71–0.82)	86.05 (81.44–90.66)	0.86 (0.79–0.91)	82.86 (76.86–88.86)	5.5 (3.5–8.6)	0.27 (0.20–0.35)	21 (11–39)	0.88 (0.84–0.90)	0.12	0.91
No-China	7	0.76 (0.65–0.84)	80.63 (66.95–94.31)	0.84 (0.74–0.91)	74.15 (54.54–93.75)	4.9 (3.0–8.1)	0.29 (0.20–0.41)	17 (10–30)	0.87 (0.84–0.90)	0.68	0.53
Specimen											
Serum	17	0.81 (0.73–0.86)	89.53 (85.64–93.41)	0.87 (0.78–0.93)	87.39 (82.45–92.33)	6.3 (3.4–11.7)	0.22 (0.15–0.32)	28 (12–69)	0.90 (0.87–0.92)	0.02	0.98
Plasma	10	0.70 (0.63–0.76)	69.60 (49.74–89.46)	0.82 (0.75–0.87)	65.85 (42.93–88.77)	3.9 (2.7–5.5)	0.37 (0.30–0.46)	10 (6–17)	0.82 (0.78–0.85)	0.15	0.89
Other specimen	5	0.78 (0.68–0.86)	68.98 (39.73–98.23)	0.90 (0.69–0.97)	72.94 (48.10–97.77)	8.0 (2.3–27.4)	0.24 (0.16–0.35)	33 (10–113)	0.88 (0.85–0.91)	0.76	0.5
Cancer type											
NSCLC	29	0.77 (0.72–0.82)	85.62 (81.19–90.06)	0.87 (0.80–0.91)	82.72 (77.10–88.34)	5.9 (3.9–9.0)	0.26 (0.21–0.33)	22 (13–40)	0.88 (0.85–0.91)	0.19	0.85
Overall	32	0.77 (0.72–0.81)	84.49 (79.83–89.14)	0.86 (0.80–0.90)	80.94 (74.89–87.00)	5.4 (3.7–7.7)	0.27 (0.22–0.34)	20 (12–33)	0.87 (0.84–0.90)	0.15	0.88

Abbreviation: *n*, number of studies.

#### Correlation between miRNA-21 expression and lung cancer

Analysis of the relationship between miRNA-21 and clinical characteristics of lung cancer revealed that expression of miRNA-21 was not significantly correlated with gender [OR = 1.04, (0.65, 1.65), *P*=0.869], smoking [OR = 1.09, (0.58, 2.05), *P*=0.784], lung cancer type [OR = 1.47, (0.92, 2.34), *P*=0.107] and lung cancer staging [OR = 0.77, (0.48, 1.22), *P*=0.263] (*P*>0.05). The results of subgroup analysis are presented in Table 6 and Supplementary Figures S25–S31.

**Table 6:** Correlation between miRNA-21 expression and clinicopathological characteristics of patients with lung cancer

Sorts	Studies	Analysis model	Participants	OR	LCI	UCI	Q	P(Q)	Z	P(Z)
Gender (Male vs Female)	5	Fixed	306	1.04	0.65	1.65	3.66	0.453	0.16	0.869
Serum	1	Fixed	218	0.89	0.38	2.07	/	/	0.26	0.792
Tissue	4	Fixed	88	1.11	0.64	1.93	3.49	0.322	0.37	0.711
Smoking history (Yes vs No)	4	Fixed	492	1.09	0.58	2.05	1.05	0.789	0.27	0.784
Tissue	3	Fixed	158	1.49	0.57	3.83	0.35	0.840	0.82	0.414
Serum	1	Fixed	88	0.85	0.36	2.00	/	/	0.36	0.716
SCC	1	Fixed	30	2.36	0.36	15.45	/	/	0.90	0.369
NSCLC	3	Fixed	216	0.98	0.50	1.93	0.31	0.856	0.05	0.959
Histological type of NSCLC (AD vs SC)	6	Fixed	354	1.47	0.92	2.34	1.57	0.904	1.16	0.107
Tissue	5	Fixed	266	1.58	0.94	2.66	1.17	0.883	1.73	0.084
Serum	1	Fixed	88	1.07	0.37	3.13	/	/	0.12	0.902
Pathological staging	6	Fixed	672	0.77	0.48	1.22	3.45	0.631	1.12	0.263
1 vs 2+	3	Fixed	168	0.77	0.37	1.62	0.14	0.934	0.68	0.496
SCC	1	Fixed	30	0.76	0.18	3.24	/	/	0.37	0.713
NSCLC	2	Fixed	138	0.78	0.33	1.85	0.14	0.713	0.57	0.566
1–2 vs 3+	3	Fixed	168	0.76	0.42	1.38	3.31	0.191	0.89	0.374
Tissue	2	Fixed	80	0.61	0.24	1.54	2.93	0.087	1.05	0.294
Serum	1	Fixed	88	0.89	0.41	1.93	/	/	0.28	0.776

Abbreviation: AC, adenocarcinoma of the lung cancer; LCI, low confidence interval; SC, lung squamous cancer; SCC, small cell lung cancer; UCI, up confidence interval; 1 vs 2+, lung cancer stage 1 vs 2–4; 1–2 vs 3+, lung cancer stage 1–2 vs 3–4.

#### Effect of miRNA-21 expression on OS of patients

A total of 14 studies [59,60,62–72] were included for univariate analysis of OS. HR (95% CI) of 1.49 (1.22–1.82) was obtained with combined analysis. High expression of miRNA-21 was associated with overall patient survival. Five studies [63,67,69–71] were included for multivariate analysis of OS. Combined HR (95% CI) of 1.65 (1.24–2.20) was obtained using a random-effects model. Our data suggest that high miRNA-21 expression is an independent risk factor for OS.

#### Effect of miRNA-21 expression on PFS/DFS of patients

A total of three studies [22,59,61] were included for PFS/DFS analysis, two of which were univariate [22,59] and two were multivariate analyses [22,61]. In the PFS/DFS univariate study, combined HR (95% CI) was 1.99 (0.73–5.43), suggesting that high expression of miRNA-21 is correlated with patient PFS/DFS. However, the data were not statistically significant (*P*=0.005).

The multivariate study using a fixed-effects model to calculate combined HR (*I^2^* = 2.5%, *P*=0.311) identified high miRNA-21 expression as an independent risk factor for PFS/DFS in patients [HR (95% CI) = 1.77 (1.19–2.62), *P*=0.004).

#### Subgroup analysis

To further reduce the possible sources of heterogeneity, we conducted different subgroup analyses by specimen, cancer type and country. Among the OS data obtained from univariate analysis, serum had the least heterogeneity, with an estimated HR value (95% CI) of 2.05 (1.61–2.60). The results are presented in Table 7 and Figure S32–S38.

**Table 7:** Subgroup analysis for miRNA-21 on the prognosis of lung cancer

Sorts	Studies	Analysis model	Participants	HR	LCI	UCI	Q	P(Q)	Z	P(Z)
Univariate										
OS	14	Fixed	1565	1.49	1.22	1.82	46.71	0.001	3.93	0.001
Specimen										
Tissue	10	Random	1240	1.25	1.03	1.51	24.40	0.004	2.27	0.023
Serum	4	Fixed	325	2.05	1.61	2.60	1.55	0.671	5.84	0.000
Cancer type										
NSCLC	10	Fixed	1315	1.65	1.21	2.26	42.36	0.000	3.15	0.002
SCC	1	Fixed	30	1.25	1.09	1.42	0.00	/	3.30	0.001
Unknown	3	Fixed	220	1.64	1.09	2.48	1.25	0.536	2.36	0.018
Country										
China	11	Fixed	798	1.60	1.30	1.97	31.93	0.000	4.45	0.000
France	1	Fixed	639	0.81	0.65	1.01	0.00	/	1.87	0.061
Athens	1	Fixed	48	1.90	0.74	4.88	0.00	/	1.33	0.182
Italy	1	Fixed	80	2.55	0.62	10.52	0.00	/	1.29	0.196
DFS/PFS	2	Fixed	138	1.99	0.73	5.43	2.14	0.143	1.34	0.181
Multivariate										
OS	5	Fixed	374	1.65	1.24	2.19	28.52	0.001	3.43	0.000
Specimen										
Tissue	4	Fixed	286	1.50	1.12	2.01	18.14	0.000	2.73	0.006
Serum	1	Fixed	88	2.01	1.49	2.72	0.00	/	4.52	0.000
Cancer type										
SCC	1	Fixed	30	1.29	1.12	1.49	0.00	/	3.57	0.000
NSCLC	4	Fixed	344	2.15	1.21	3.80	28.06	0.000	2.62	0.009
DFS/PFS	2	Fixed	109	1.78	1.18	2.66	1.02	0.310	2.76	0.005
Specimen										
Tissue	1	Fixed	58	2.82	1.05	7.55	0.00	/	2.06	0.039
Serum	1	Fixed	51	1.62	1.06	2.48	0.00	/	2.21	0.027

Abbreviations: LCI, low confidence interval; SCC, small cell lung cancer; UCI, up confidence interval.

#### Publication bias

Begg’s and Egger’s tests were applied to assess publication bias. Since less than ten articles were included in multivariate analysis of OS and univariate/multivariate analysis of PFS/DFS [73], we only performed publication bias analysis on literature included in the OS univariate analysis. The value obtained with Begg’s test was 0.77 (*P*=0.44) and that with Egger’s test was 3.43 (*P*=0.005), indicating potential publication bias. In subgroup analysis by serum and tissue, Begg’s test results were 0.34 (*P*=0.734) and 1.07 (*P*=0.283) and Egger’s test results were 2.49 (*P*=0.350) and 1.86 (*P*=0.520), respectively. The data suggest no obvious publication bias and good credibility of subgroup analysis.

## Discussion

Following introduction of the concept of miRNAs in 2001, thousands of these molecules have been identified in the human genome [74]. MiRNAs exist stably in blood and plasma with the majority losing their normal regulatory mechanisms in cancer cells. With the continuous advancements in science and technology, numerous differentially expressed miRNAs have been identified in miRNA profile analyses of lung cancer cells through real-time PCR [22], which are considered potential targets for diagnosis, treatment and prognosis [75]. One of the first publications to demonstrate a possible role of miR-21 as a prognostic factor in NSCLC was documented by Markou et al. (2008) [69]. The group reported up-regulated miRNA levels in tumor tissue compared with their paired normal control counterparts. In 2013, Pereira et al. [76] proposed that miRNA-21 acts as a key post-transcriptional regulator with utility as a tumor-specific biomarker for cancer diagnosis, prognosis and treatment responses, which since been comprehensively investigated in patients with lung cancer. Previously, our group showed that miRNA-21 is consistently highly expressed in lung cancer tissues [77] and promotes the occurrence of lung cancer and migration of tumor cells by inhibiting negative regulators of the RAS/MEK/ERK and MAPK/ERK signaling pathways and expression of KIBRA [77,78]. These earlier studies collectively support the efficacy of miRNA-21 as a biomarker for lung cancer [79,80]. However, due to limited sample sizes or large sample variability among studies, expression of miRNA-21 in whole blood and peripheral blood cells of lung cancer patients did not appear significantly different from those of healthy controls in some analyses. Such as the study of Meng et al., they said the evaluation of miRNA-21 expression was proposed to be ineffective for early diagnosis of lung cancer [81]. In the current study, we conducted a meta-analysis for systematic assessment of the correlation between miRNA-21 and lung cancer and its clinical value in the early diagnosis and prognosis of the disease.

### Diagnostic meta-analysis

The current meta-analysis was performed with the aim of determining the precise role of miRNA-21 in lung cancer. Through analysis of 32 included studies [14,15,30–58], the sensitivity of miRNA-21 in lung cancer diagnosis was determined as 0.77 (95% CI = 0.72–0.81), specificity as 0.86 (95% CI = 0.80–0.90), and AUC as 0.87 (95% CI = 0.84–0.90). These three representative parameters validated the diagnostic accuracy of miRNA-21 in patients with lung cancer. A DOR value of 20 (95% CI = 12–33) was obtained, further supporting the utility of miRNA-21 as a valuable marker for lung cancer. Our findings are consistent with data from large-scale studies by the groups of Xiaoqin [40] and Jing [31], Yongpan [54] and other researchers. The maximum sensitivity was 0.932 and specificity was 0.964. A study by Yang et al. [30] was considered the main source of heterogeneity and had a significant impact. However, after reviewing all the included literature, this study [30] was considered immensely valuable. The group confirmed that expression of miRNA-21 was positively correlated with clinical stage of NSCLC and concluded that, if necessary, miRNA-21 evaluation should be recommended as the first choice to aid in diagnosis and clinical staging. After excluding individual studies through sensitivity analysis, the results obtained upon re-analysis showed no significant changes and further confirmed the robustness of diagnostic meta-analysis results and value of miRNA-21 in diagnosis. Accumulating research additionally suggests that miRNA-21 exerts effects similar to oncogenes and tumor suppressor genes [82]. MiRNAs degrade or inhibit mRNA translation by binding the untranslated region of target mRNAs, thereby participating in related regulation of cell growth, development, proliferation, differentiation and apoptosis. A number of studies support the tumor-promoting effect of SIRT1 and positive correlation of miRNA-21 with SIRT1 expression [62]. Findings to date indicate that SIRT1 in lung cancer is indirectly regulated by miRNA-21 but the precise mechanisms of action remain to be established. These studies provide a potential direction for treatment of lung cancer via targeting of miRNA-21.

### Prognostic meta-analysis

High expression of miRNA-21 in was significant in prognosis of lung cancer, which could be used to predict recurrence and risk of adverse reactions [83]. We observed no correlations between abnormal expression of miRNA-21 and gender, smoking habits, pathological type or clinical stage of lung cancer in the included studies. Notably, our conclusions on the relationship between miRNA-21 and pathological type and clinical stage were inconsistent with those of Haigl et al. [76]. The latter study showed that increase in miRNA-21 expression was correlated with malignancy of lung cancer tissues and expression in adenocarcinoma was higher than that in squamous cell carcinoma tissues. The high proportion of negative results in our correlation analysis may account for this discrepancy. Therefore, the specific correlations between miRNA-21, pathological type and clinical stage require further investigation.

Expression of miRNA-21 in lung cancer tissues with higher pathological grade is reported to be markedly higher relative to that in samples with lower pathological grade [84]. Moreover, high miRNA-21 expression is a significant independent risk factor for patients with positive lymph node metastasis [85]. In lung cancer, overexpression of miRNA-21 is closely related to adverse prognostic events [69]. However, inconsistent with these findings, miRNA-21 could not be used to predict prognosis in a study by Voortman et al. [86]. To resolve the controversy, the current meta-analysis was conducted to evaluate the value of miRNA-21 in prognosis of lung cancer. The results of univariate analysis indicate that miRNA-21 is a risk factor for lung cancer prognosis (HR = 1.49, 95% CI: 1.22–1.82). Consistently, results obtained from multivariate analysis (HR = 1.65, 95% CI: 1.24–2.19) validated miRNA-21 as an independent risk factor for poor prognosis of lung cancer patients. The collective results support the efficacy of miRNA-21 as a biomarker of prognosis of lung cancer that may be appropriate for clinical evaluation of survival risk of patients. Highly expressed miRNA-21 is proposed to directly increase tumor cell proliferation and invasion through targeting of the *PTEN* gene [87]. Abnormal expression of miRNA-21 is closely related to the onset and prognosis of a variety of tumors and acts as a tumor ‘oncogene’ regulator with a critical role in prognosis [88]. Detection of miRNA-21 in patients with lung cancer may therefore aid in assessment of disease progression and prognosis of patients to provide appropriate guidelines for clinical treatment.

### Limitations

Our research has a number of limitations that should be taken into consideration. Firstly, although we attempted to search all miRNA-21-related literature on diagnosis and prognosis of lung cancer and included a large number of samples, some valuable studies may have been overlooked. In addition, all the included studies showed positive results, which could indicate that negative results were not published. Secondly, we only included Chinese and English literature, and therefore, important research published in other languages were missing and a certain language deviation existed. At the same time, this meta-analysis was not universal for non-Chinese and English-speaking countries. While heterogeneity across studies was inevitable (race, follow-up time), we additionally performed subgroup analysis of the clinical characteristics of patients (ethnicity, country, lung cancer type) and specimen types (serum, plasma, tissue). Fourthly, there was no mention of blinding in some diagnostic studies, which could affect the reliability of miRNA-21 in diagnosis of lung cancer.

## Conclusion

In conclusion, the sensitivity and specificity of miRNA-21 in early diagnosis of lung cancer needs further consideration and its diagnostic value remains to be established. However, overexpression of miRNA-21 is strongly correlated with poor prognosis of patients, supporting its utility as a prognostic biomarker of lung cancer. Further prospective studies with larger sample sizes are required to facilitate early diagnosis, clinical staging and prognosis evaluation of lung cancer.

## Supplementary Material

Supplementary Figures S1-S38

## Data Availability

The datasets analyzed in the present study are available from the published papers that have been cited in this manuscript.

## Abbreviations

AUC: area under the summary receiver operating characteristic (SROC) curve
CI: confidence interval
DFS: disease-free survival
DOR: diagnostic odds ratio
HR: hazards ratio
miRNA-21: microRNA-21
NLR: negative likelihood ratio
NOS: Newcastle–Ottawa Scale
NSCLC: non-small cell lung cancer
OR: odds ratio
OS: overall survival
PFS: progression-free survival
PLR: positive likelihood ratio
QUADAS-2: Quality Assessment of Diagnostic Accuracy Studies-2
RR: relative risk
SCLC: small-cell lung cancer
SEN: sensitivity
SPE: specificity
