# Supplementary material for: MicroRNA-21 as a diagnostic and prognostic biomarker of lung cancer: a systematic review and meta-analysis
Source: Biosci Rep. 2022 May 10;42(5):BSR20211653. doi: 10.1042/BSR20211653 (PMC9093699; doi:10.1042/BSR20211653)
Supplement: Supplementary Figures S1-S38 [file BSR-2021-1653_supp.pdf]

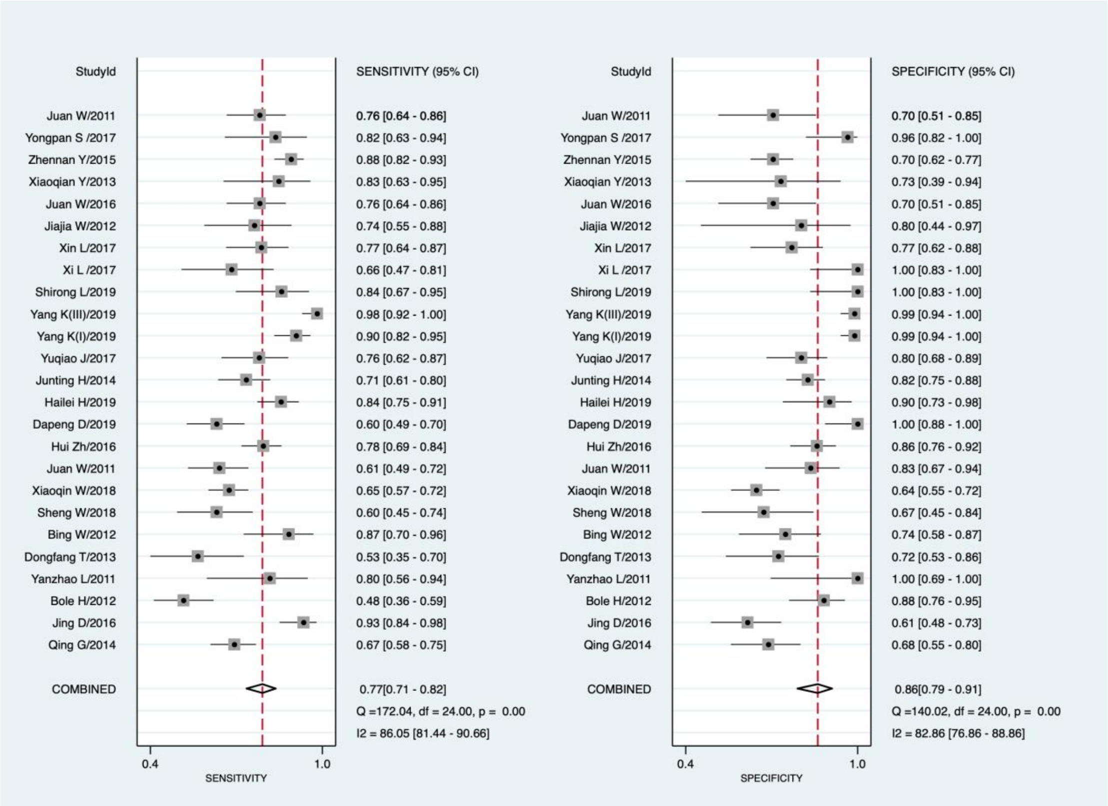

Figure S1. Forest plot of sensitivity and specificity of miRNA-21 in diagnosis of lung cancer (China).

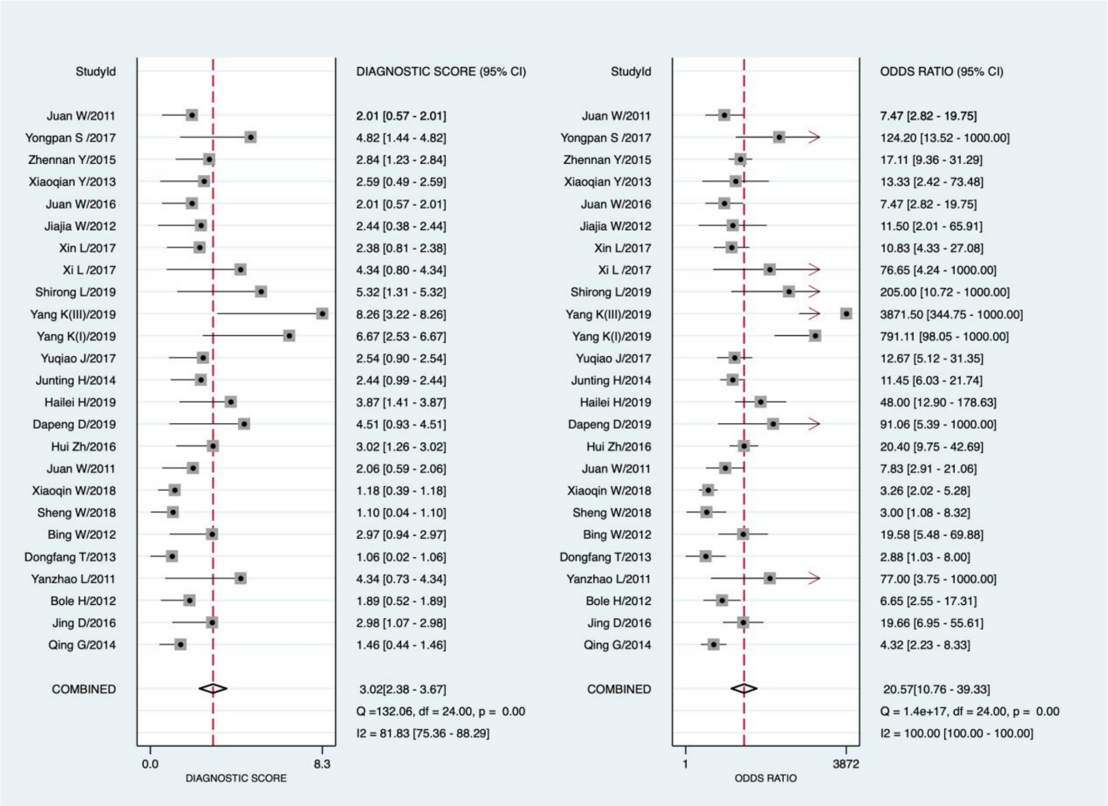

Figure S2. Forest plots of odds ratio of miRNA-21 in diagnosis of lung cancer (China).

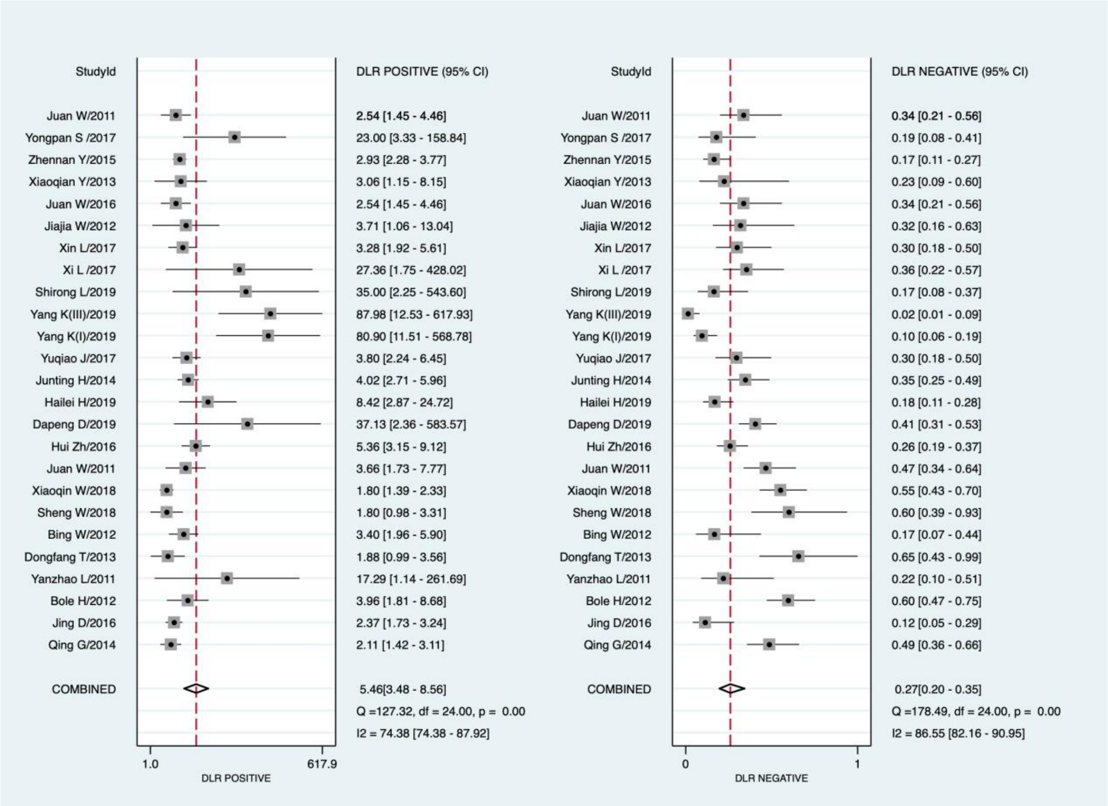

Figure S3. Forest plots of DLR positivity and DLR negativity of miRNA-21 in diagnosis of lung cancer (China).

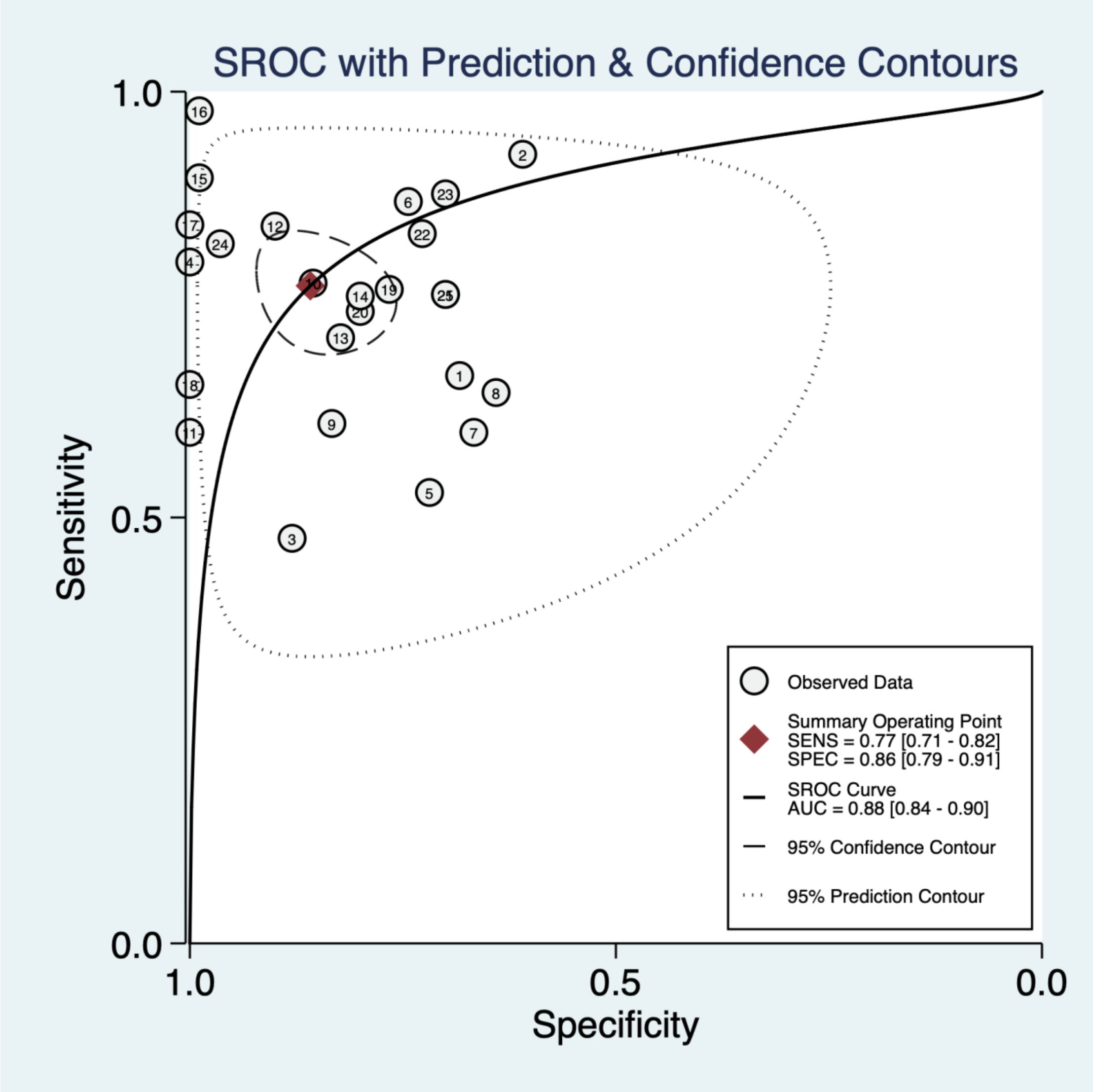

Figure S4. SROC curves of miRNA-21 for diagnosis of lung cancer (China).

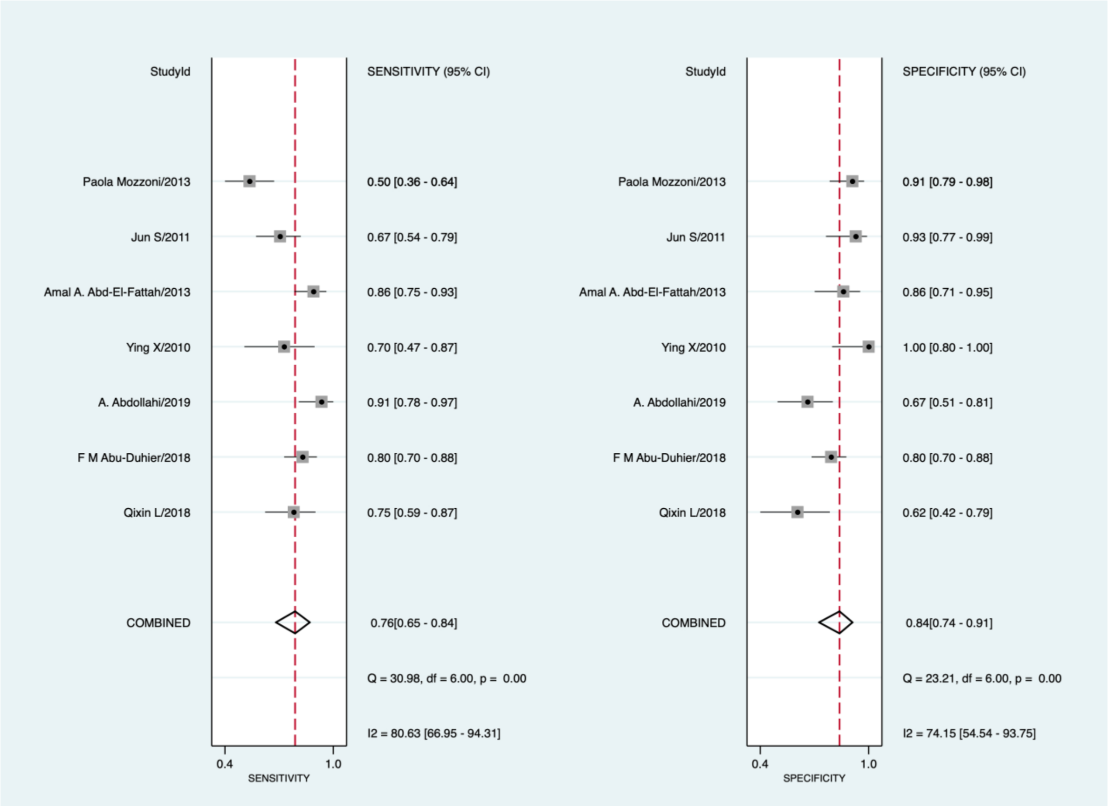

Figure S5. Forest plot of sensitivities and specificities of miRNA-21 in diagnosis of lung cancer (Non-China).

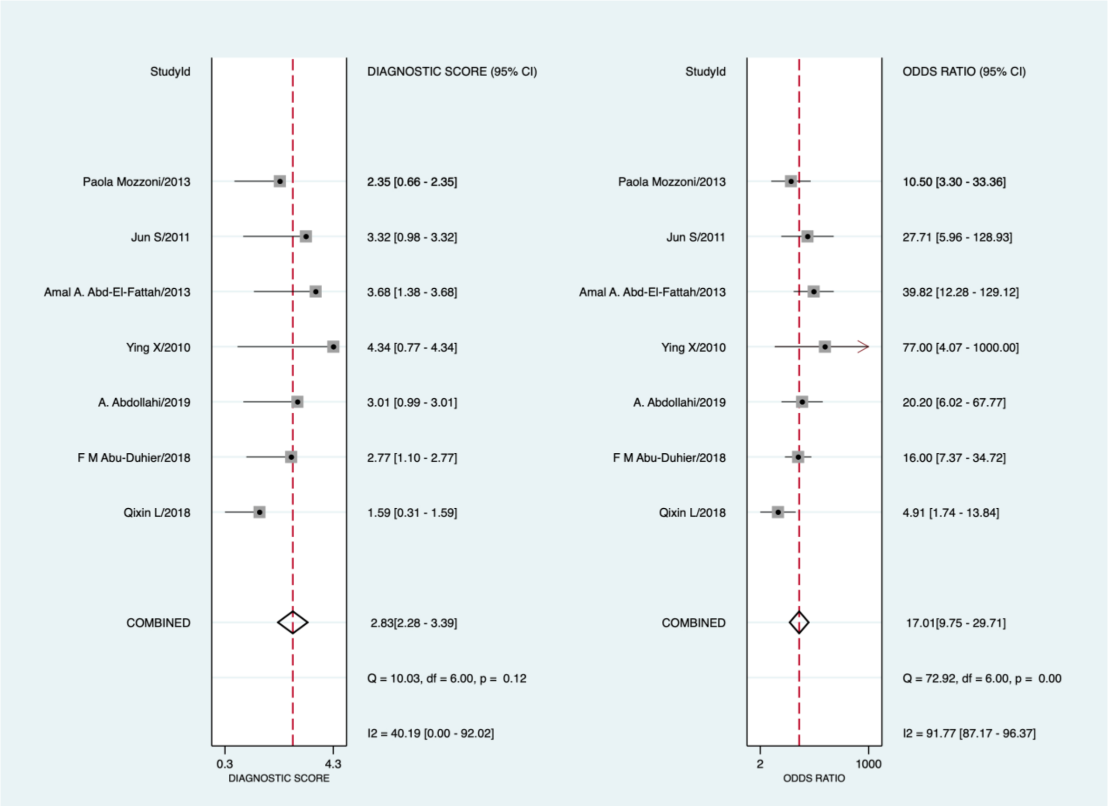

Figure S6. Forest plots of odds ratio of miRNA-21 in diagnosis of lung cancer (Non-China).

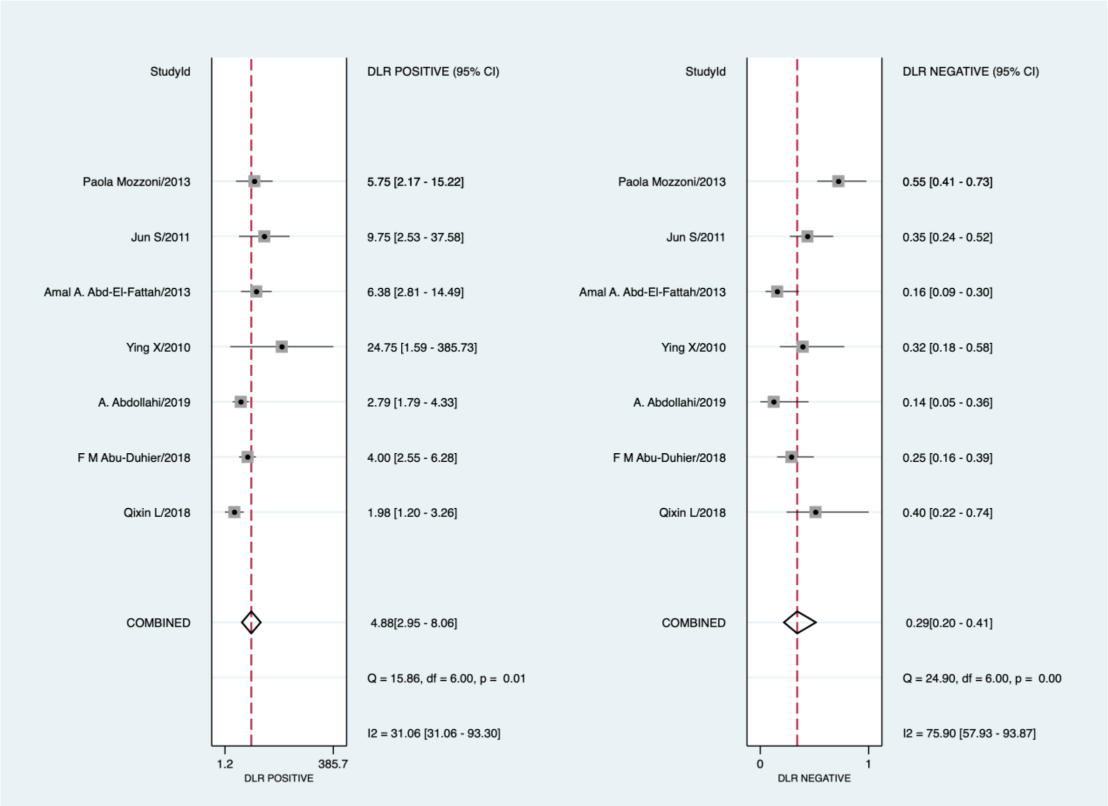

Figure S7. Forest plots of DLR positivity and DLR negativity of miRNA-21 in diagnosis of lung cancer (Non-China).

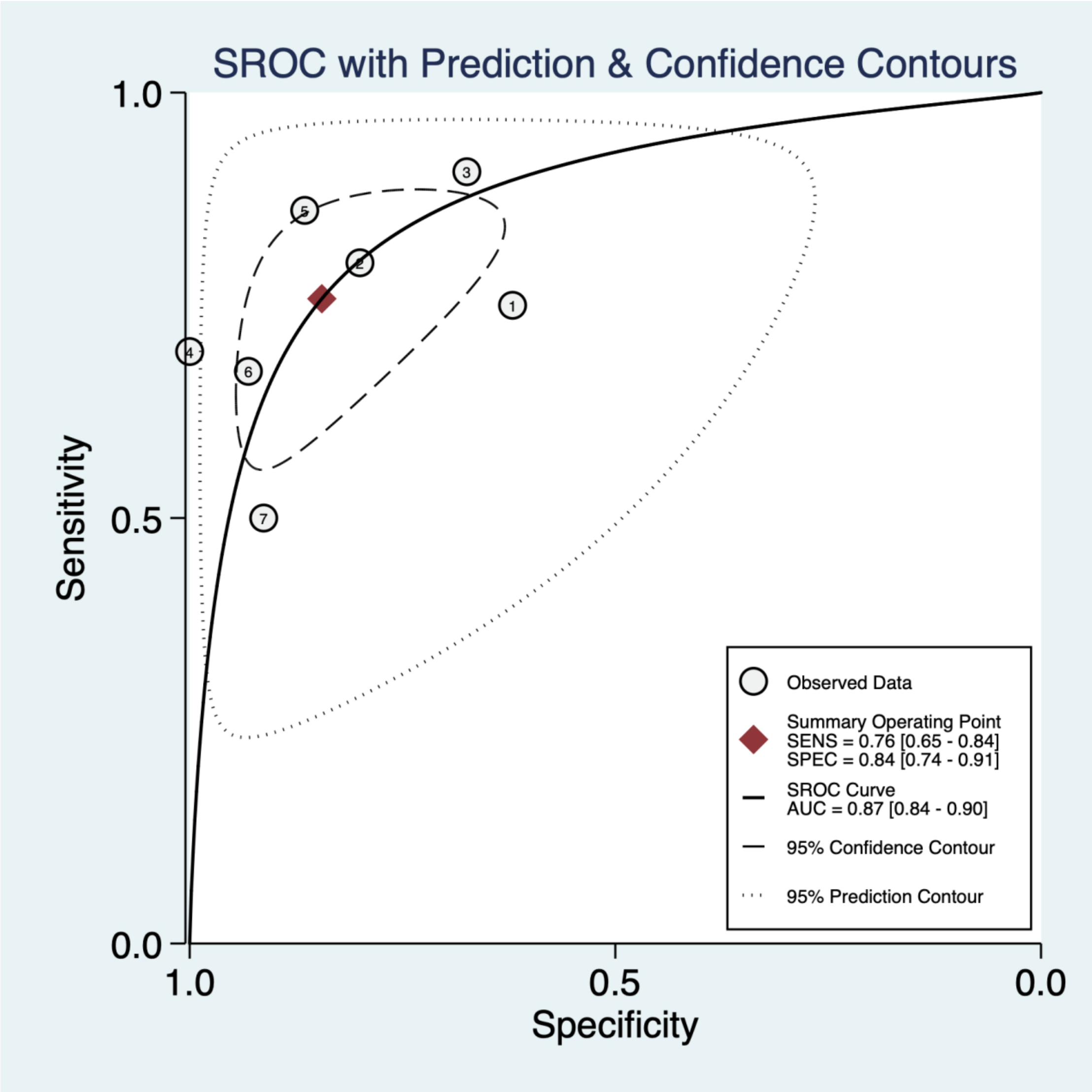

Figure S8: SROC curves of miRNA-21 for diagnosis of lung cancer (Non-China).

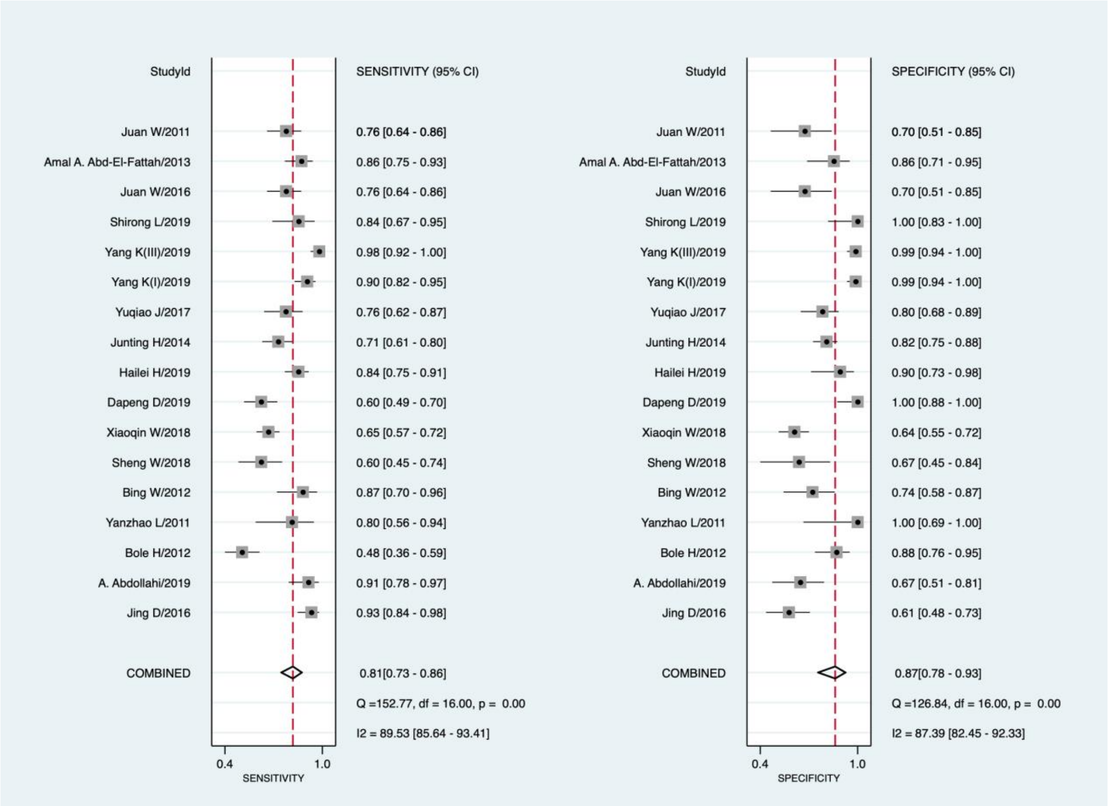

Figure S9. Forest plot of the sensitivities and specificities of miRNA-21 in diagnosis of lung cancer (Serum).

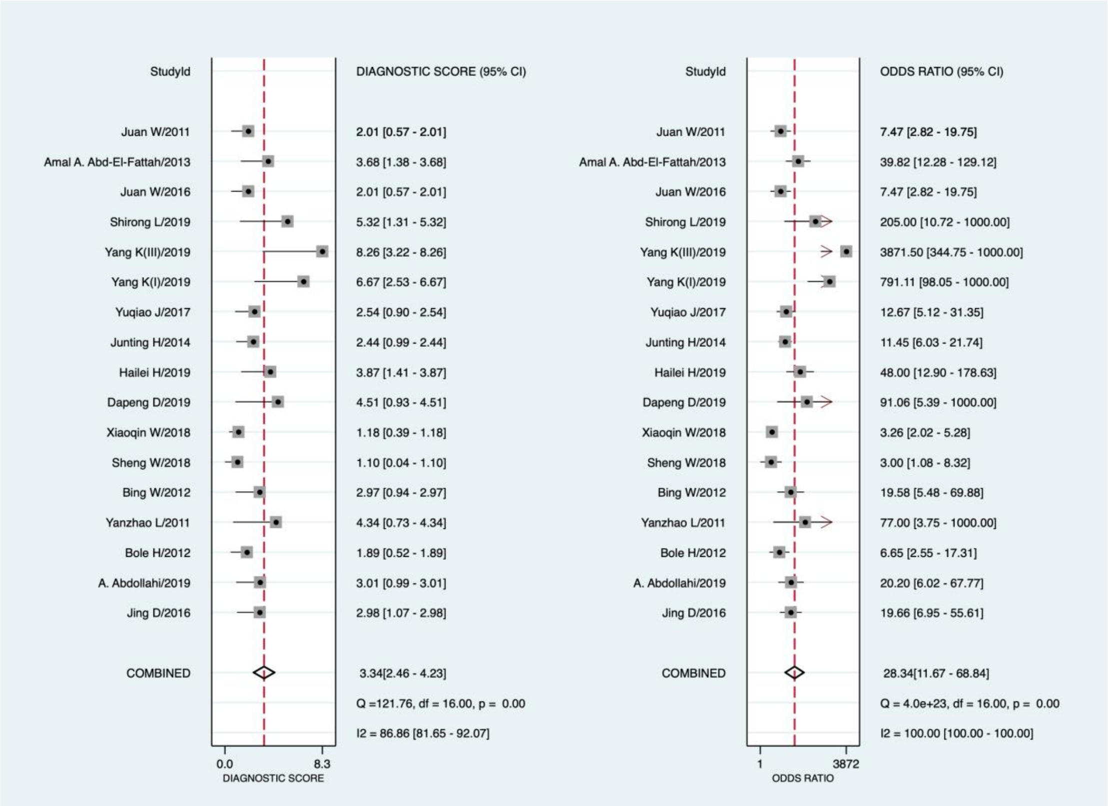

Figure S10. Forest plots of odds ratio of miRNA-21 in diagnosis of lung cancer (Serum).

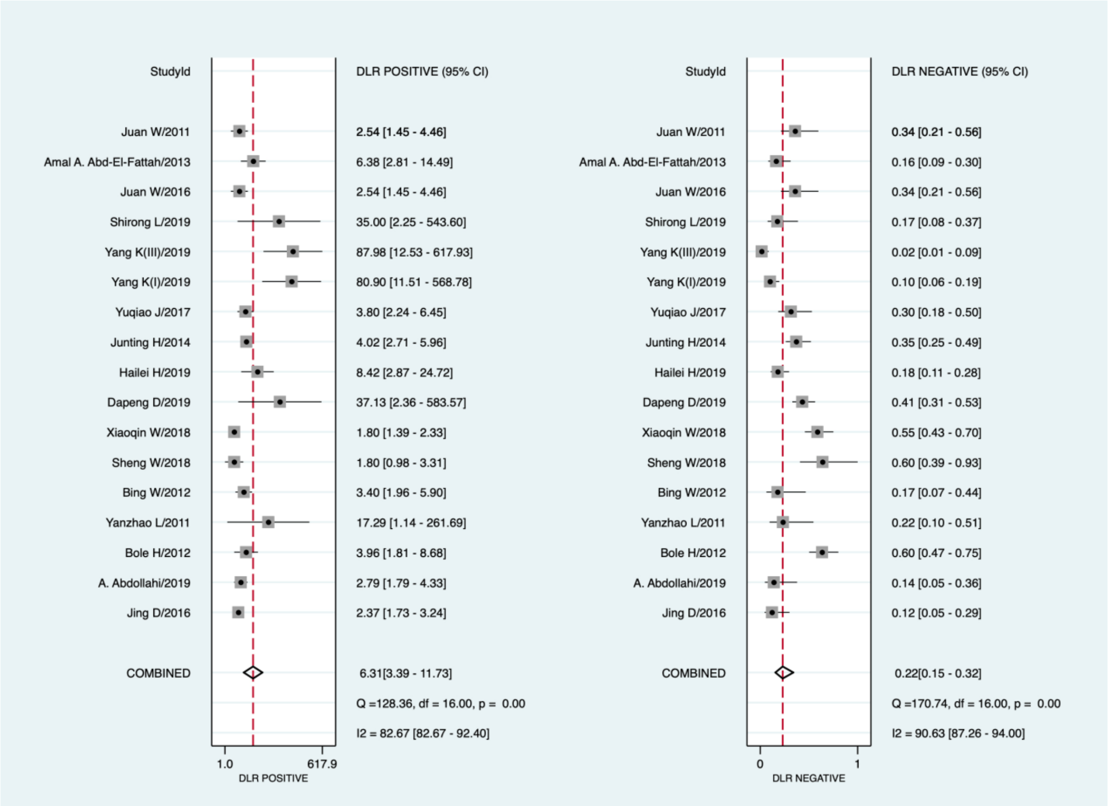

Figure S11. Forest plots of DLR positivity and DLR negativity of miRNA-21 in diagnosis of lung cancer (Serum).

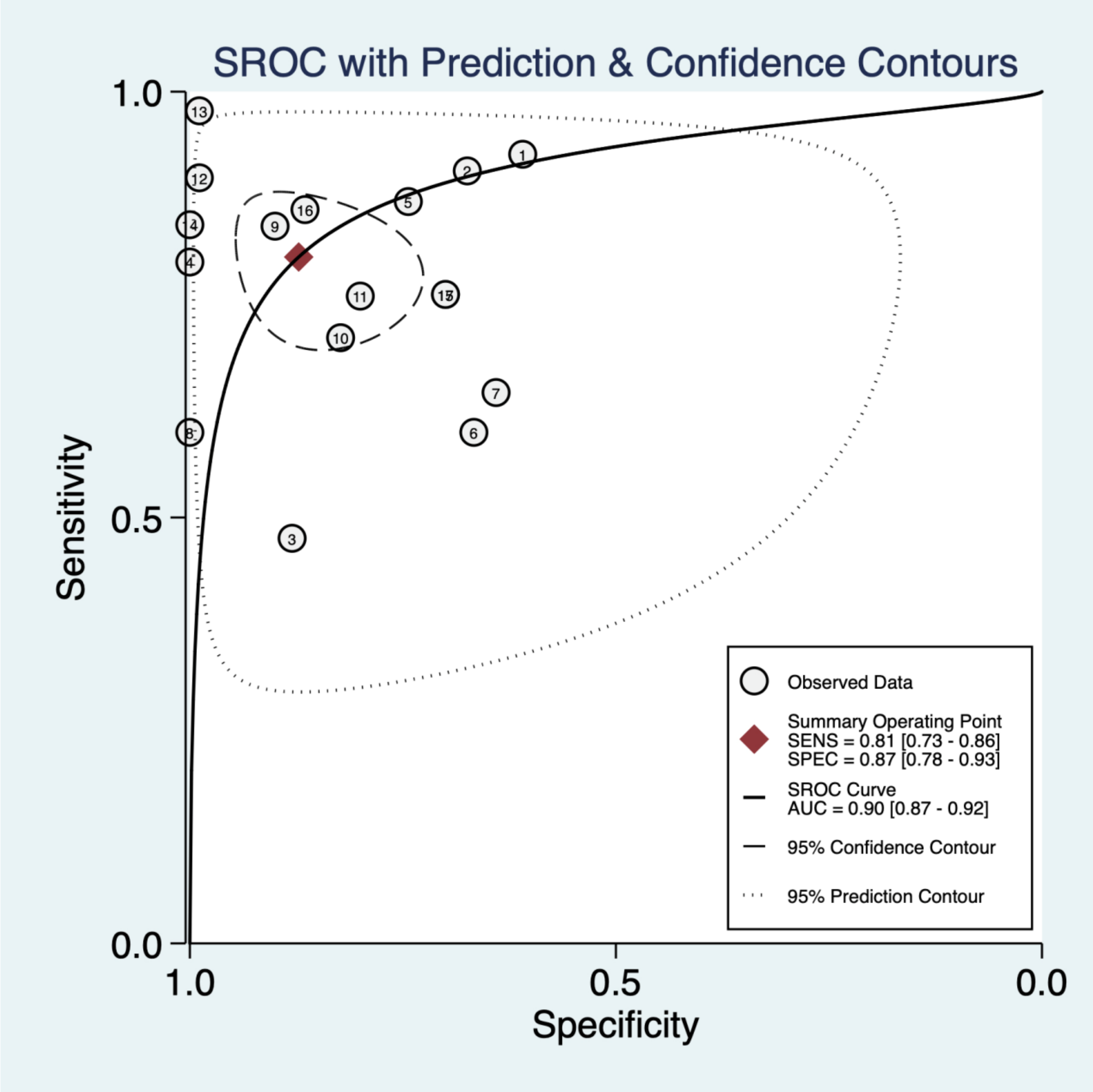

Figure S12. SROC curves of miRNA-21 in diagnosis of lung cancer (Serum).

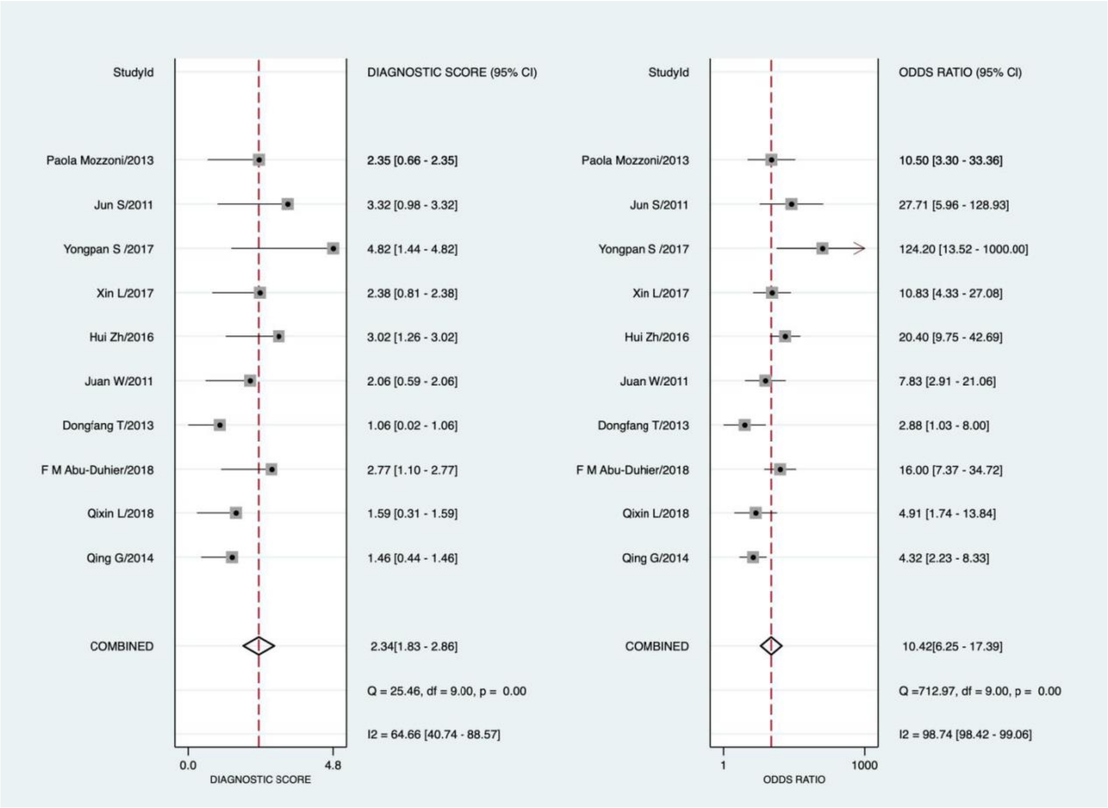

Figure S13. Forest plot of sensitivities and specificities of miRNA-21 in diagnosis of lung cancer (Plasma).

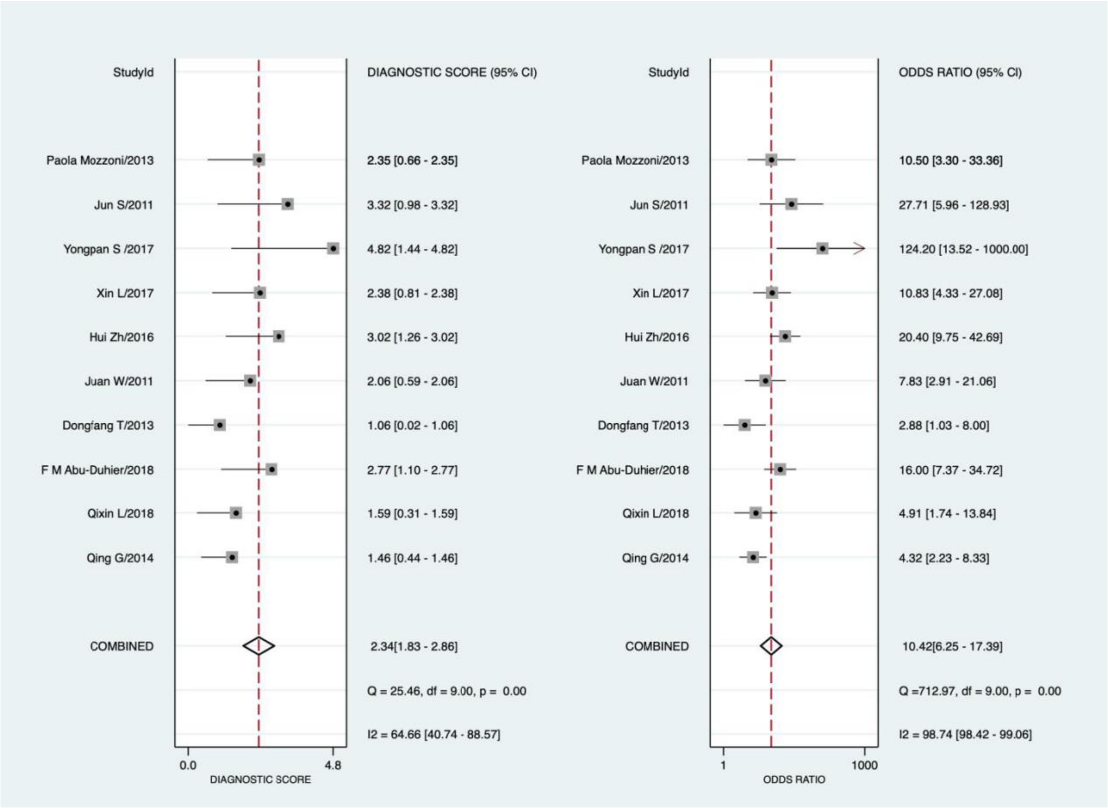

Figure S14. Forest plots of odds ratio of miRNA-21 in diagnosis of lung cancer (Plasma).

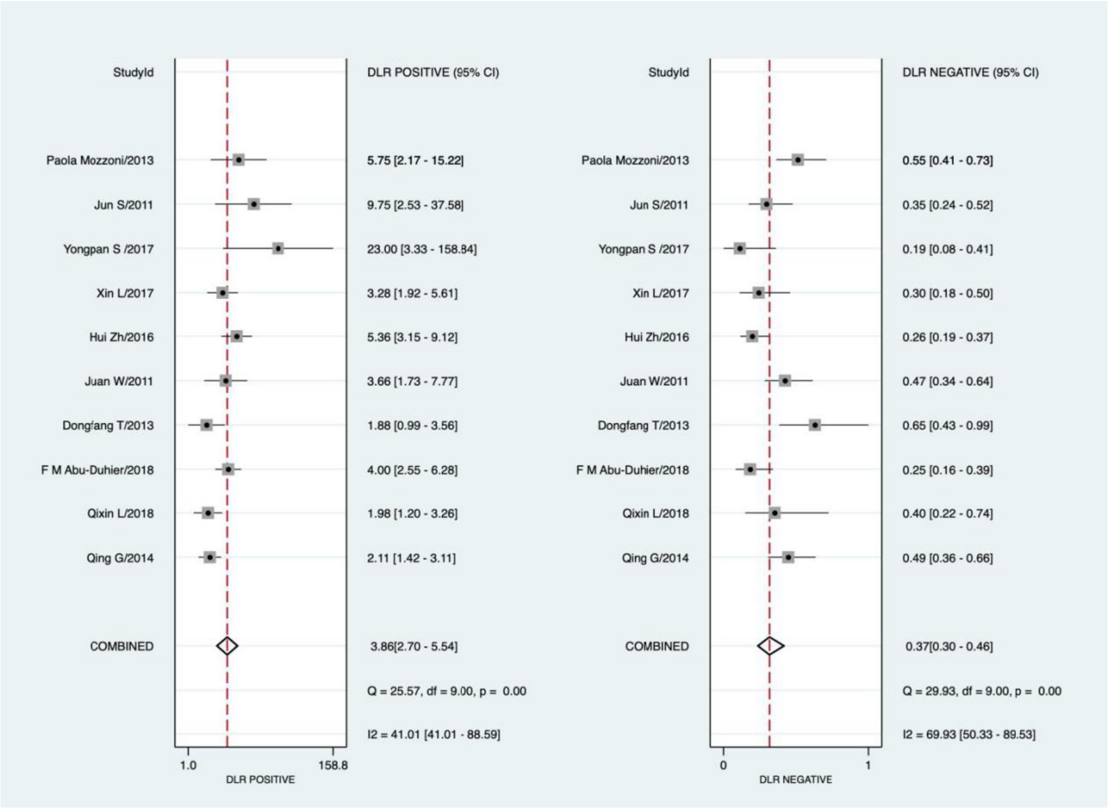

Figure S15. Forest plots of DLR positivity and DLR negativity of miRNA-21 in diagnosis of lung cancer (Plasma).

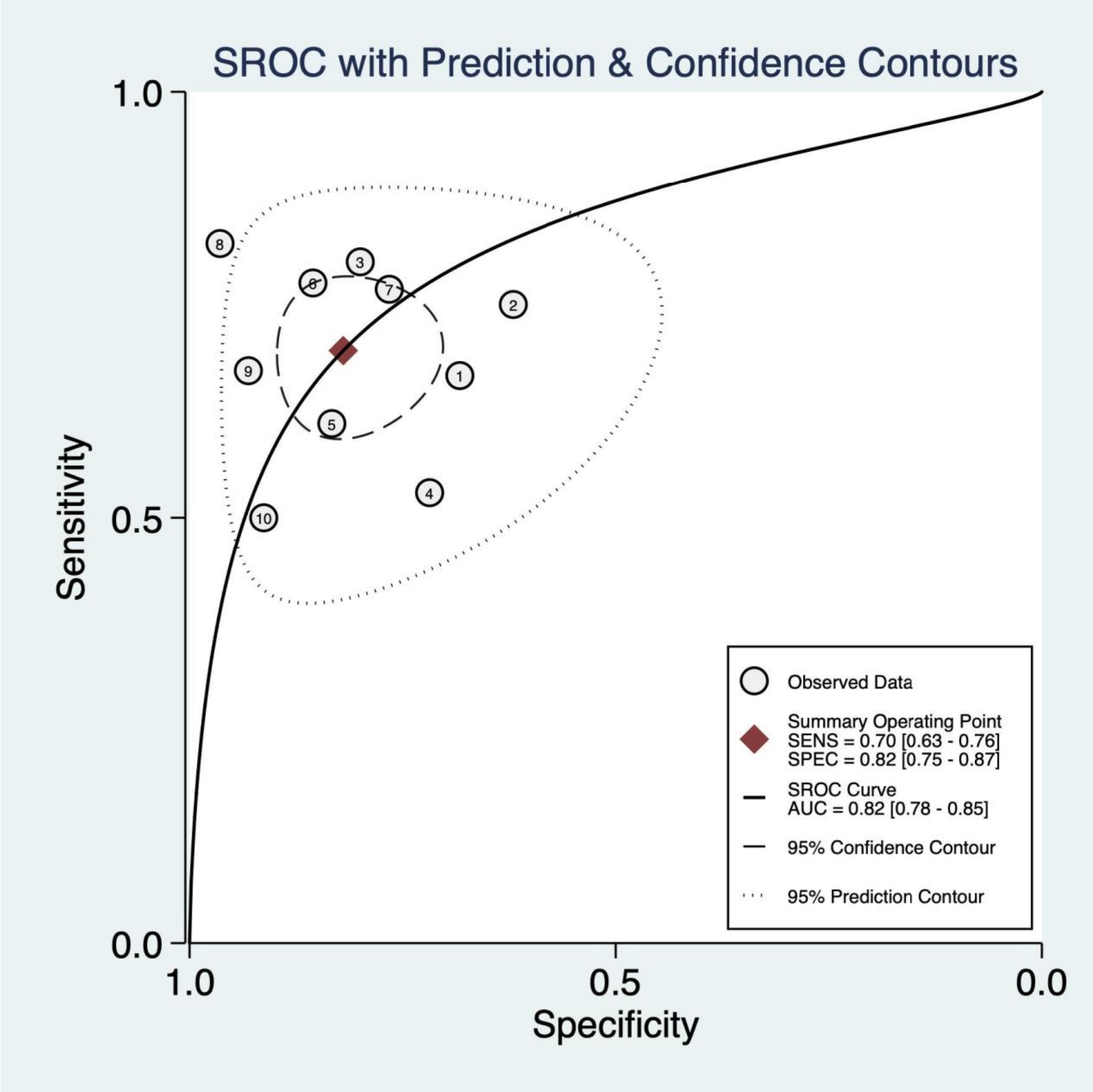

Figure S16. SROC curves of miRNA-21 for diagnosis of lung cancer (Plasma).

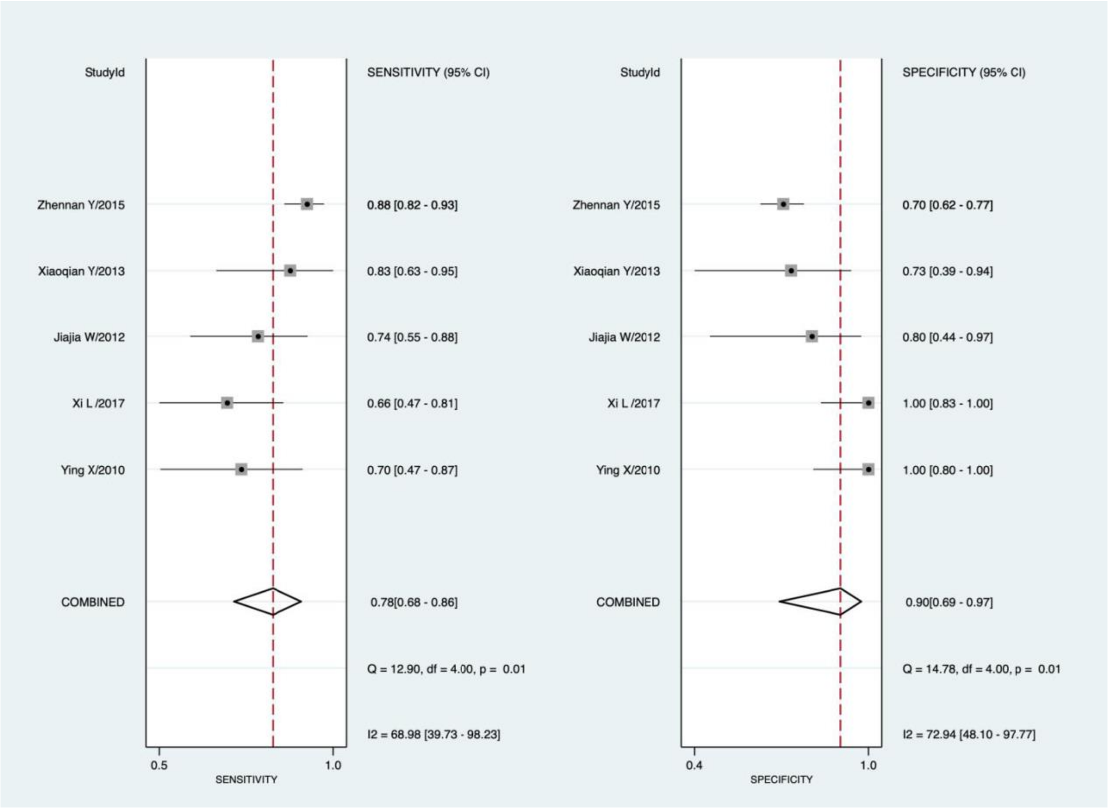

Figure S17. Forest plot of sensitivities and specificities of miRNA-21 in diagnosis of lung cancer (Other specimens).

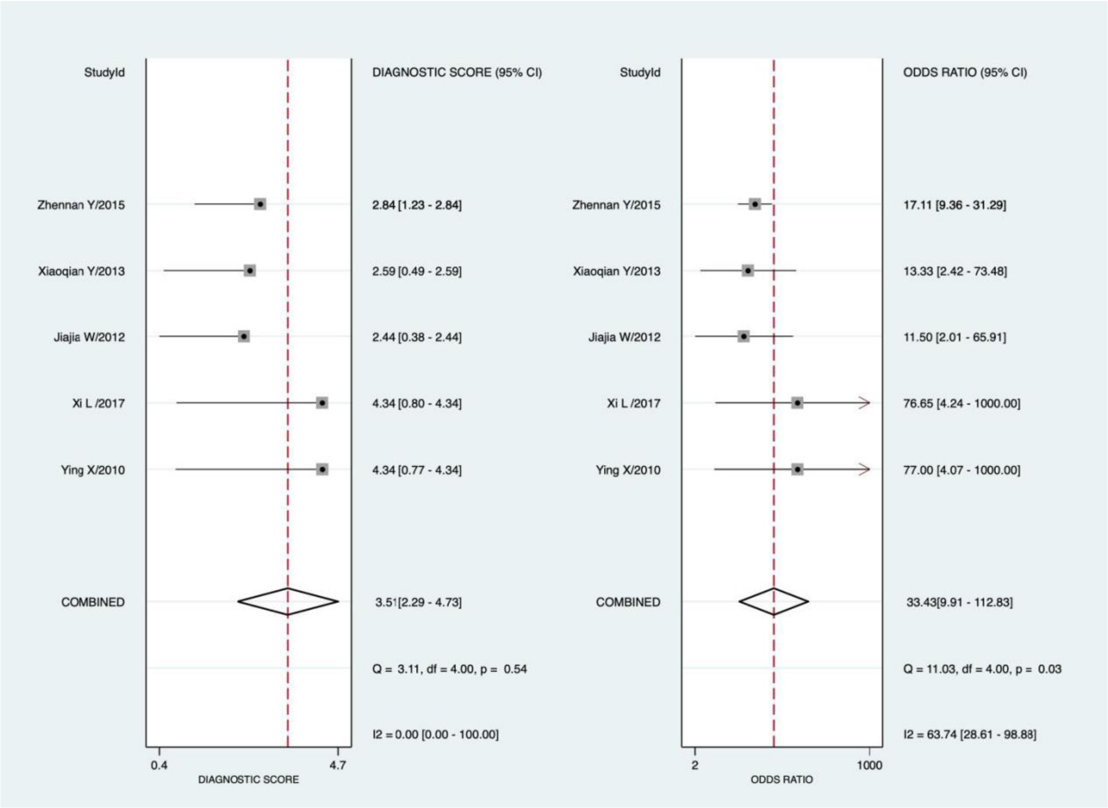

Figure S18. Forest plots of odds ratio of miRNA-21 in diagnosis of lung cancer (Other specimens).

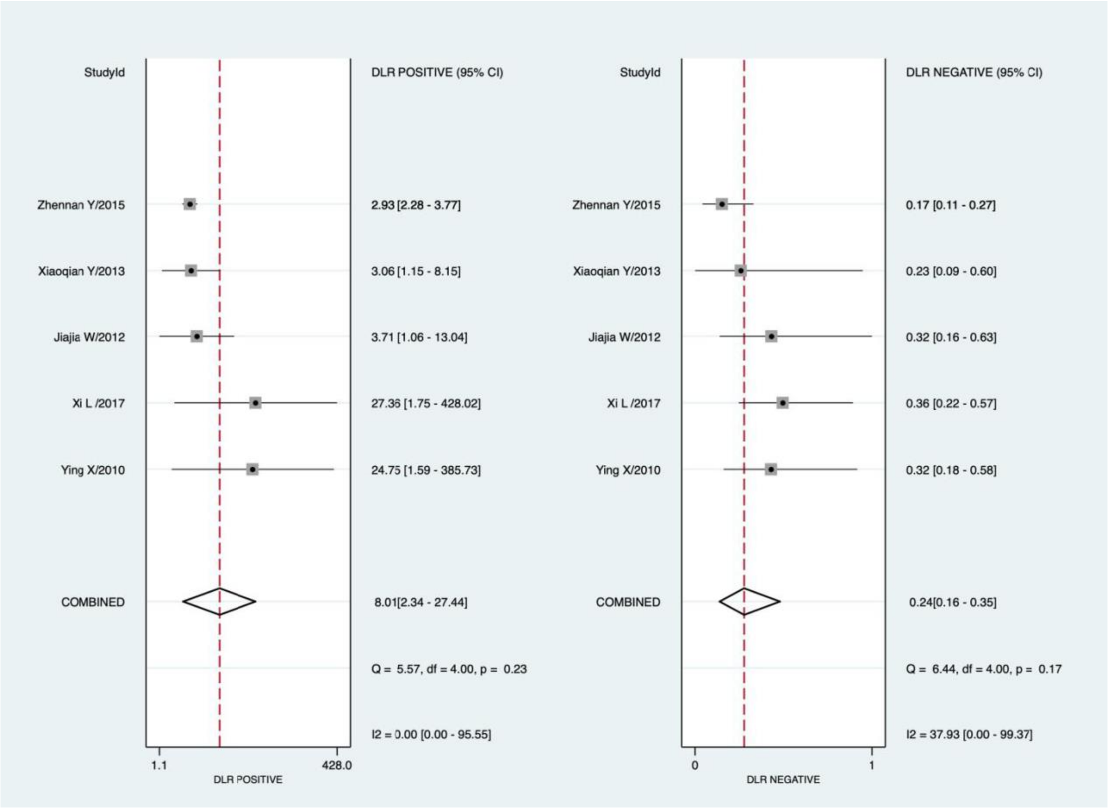

Figure S19. Forest plots of DLR positivity and DLR negativity of miRNA-21 in diagnosis of lung cancer (Other specimens).

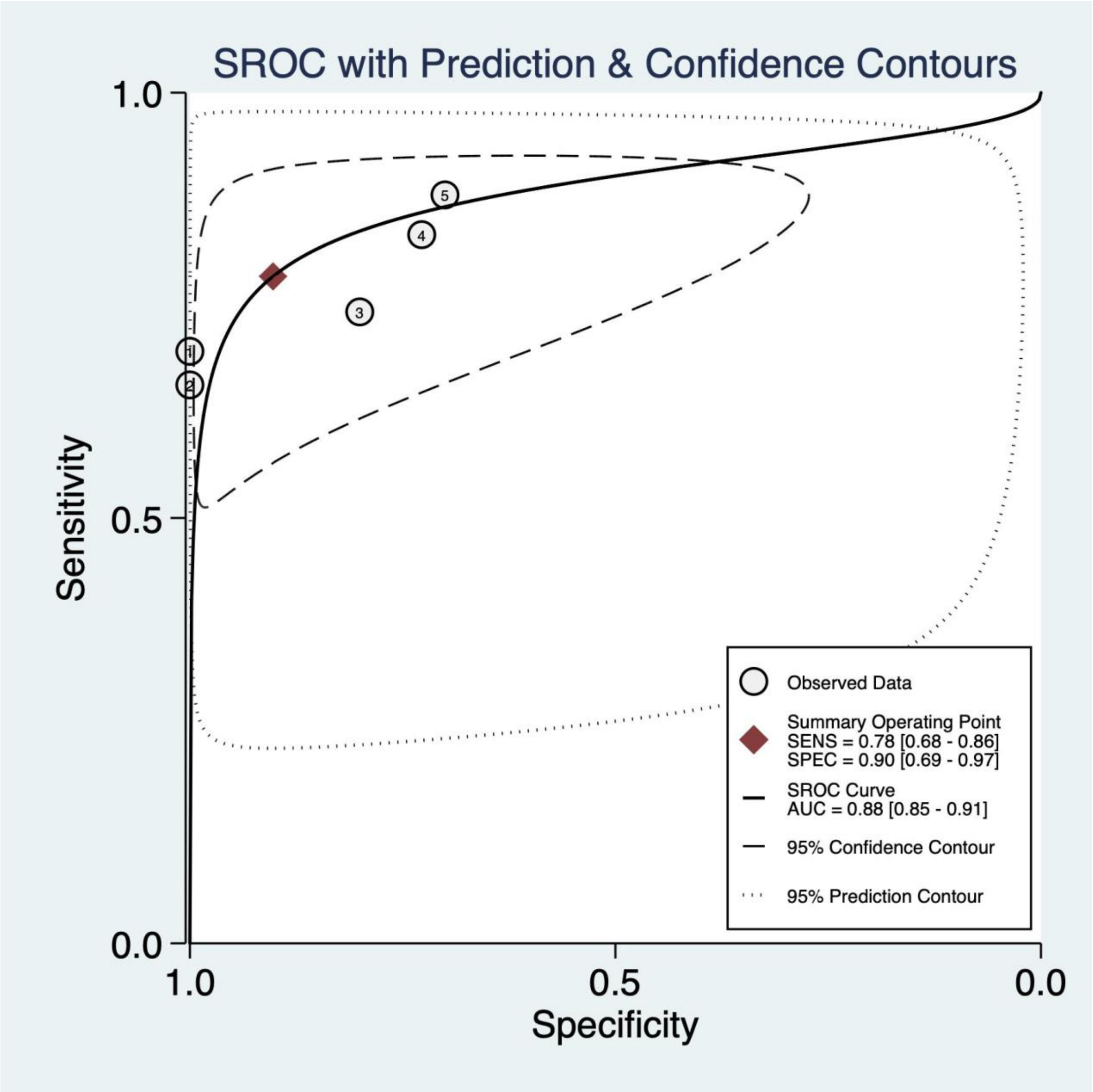

Figure S20. SROC curves of miRNA-21 for diagnosis of lung cancer (Other specimens).

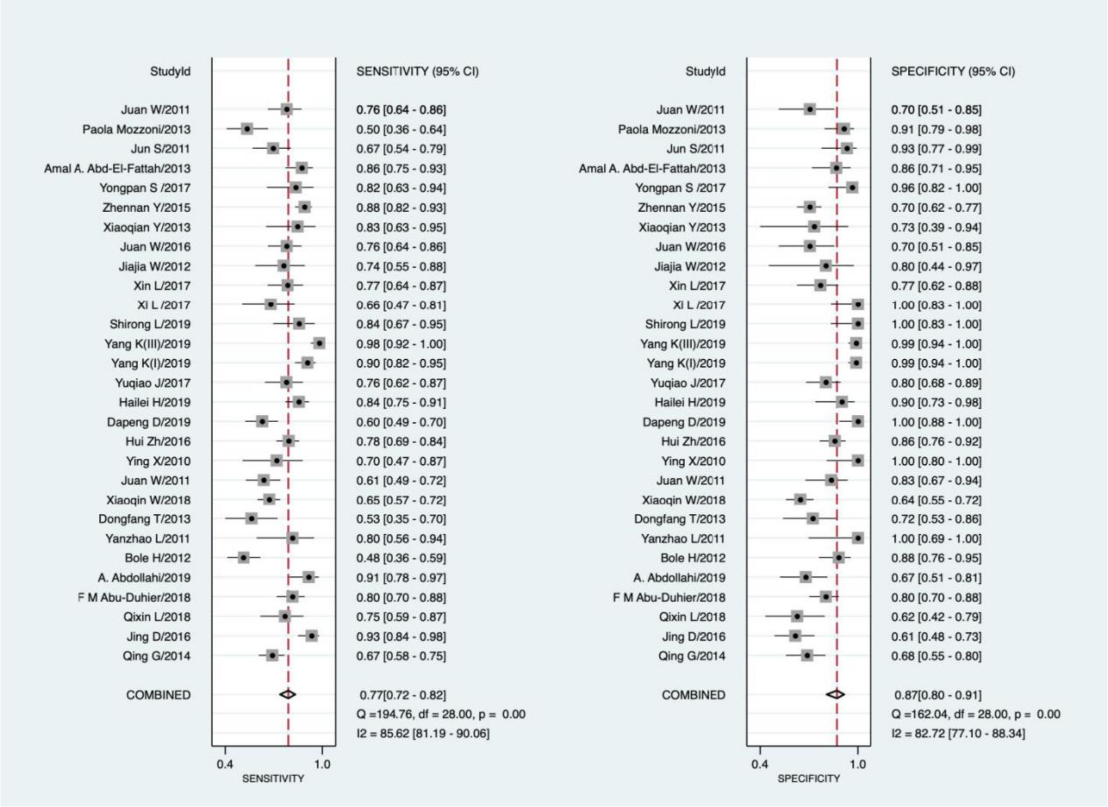

Figure S21. Forest plot of sensitivities and specificities of miRNA-21 in diagnosis of lung cancer (NSCLC).

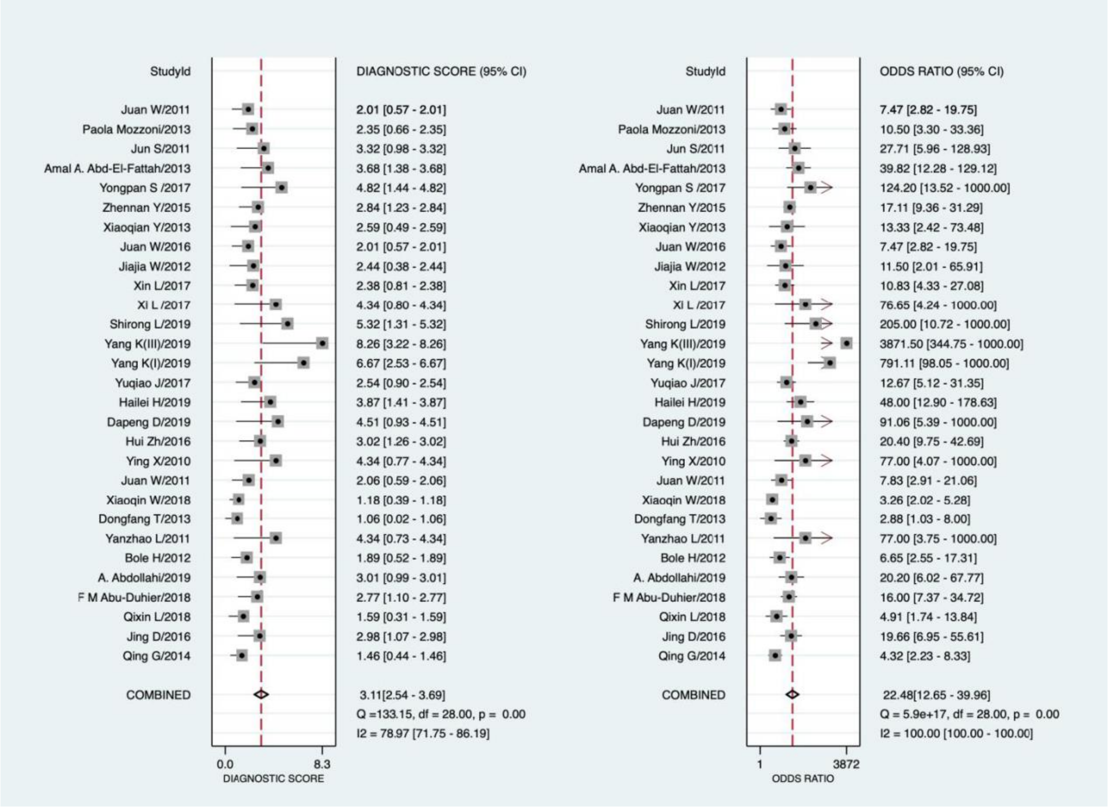

Figure S22. Forest plots of odds ratio of miRNA-21 in diagnosis of lung cancer (NSCLC).

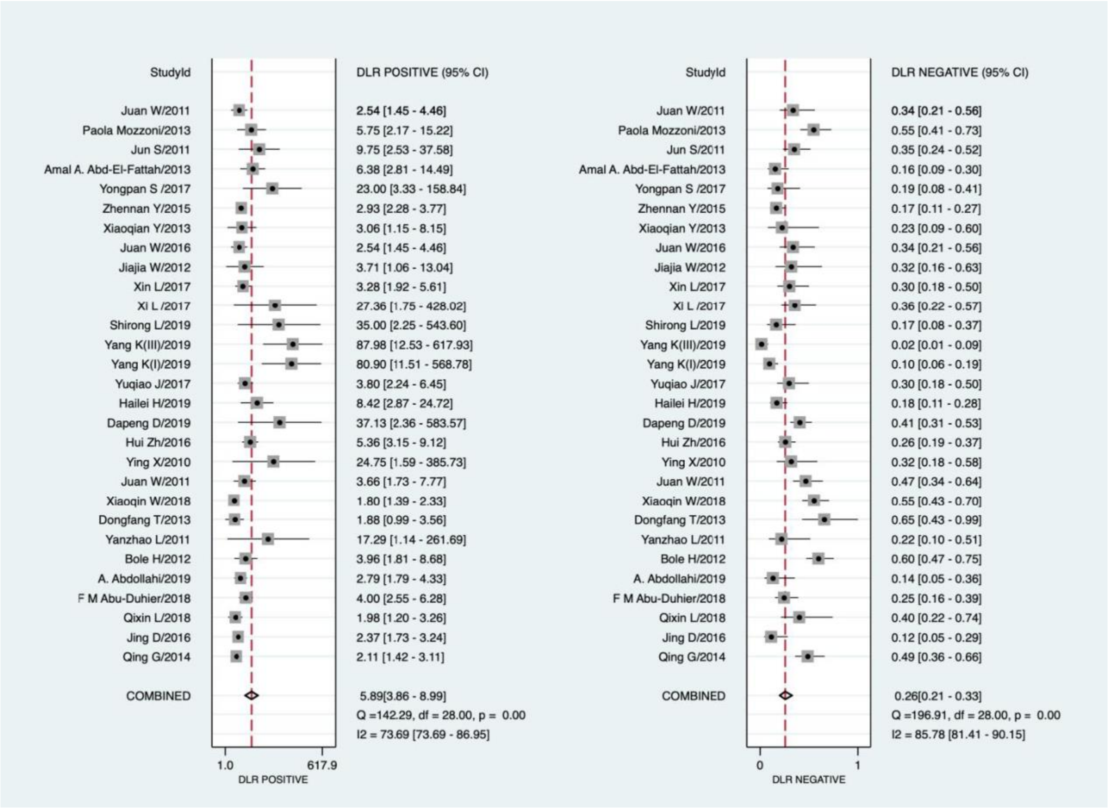

Figure S23. Forest plots of DLR positivity and DLR negativity of miRNA-21 in diagnosis of lung cancer (NSCLC).

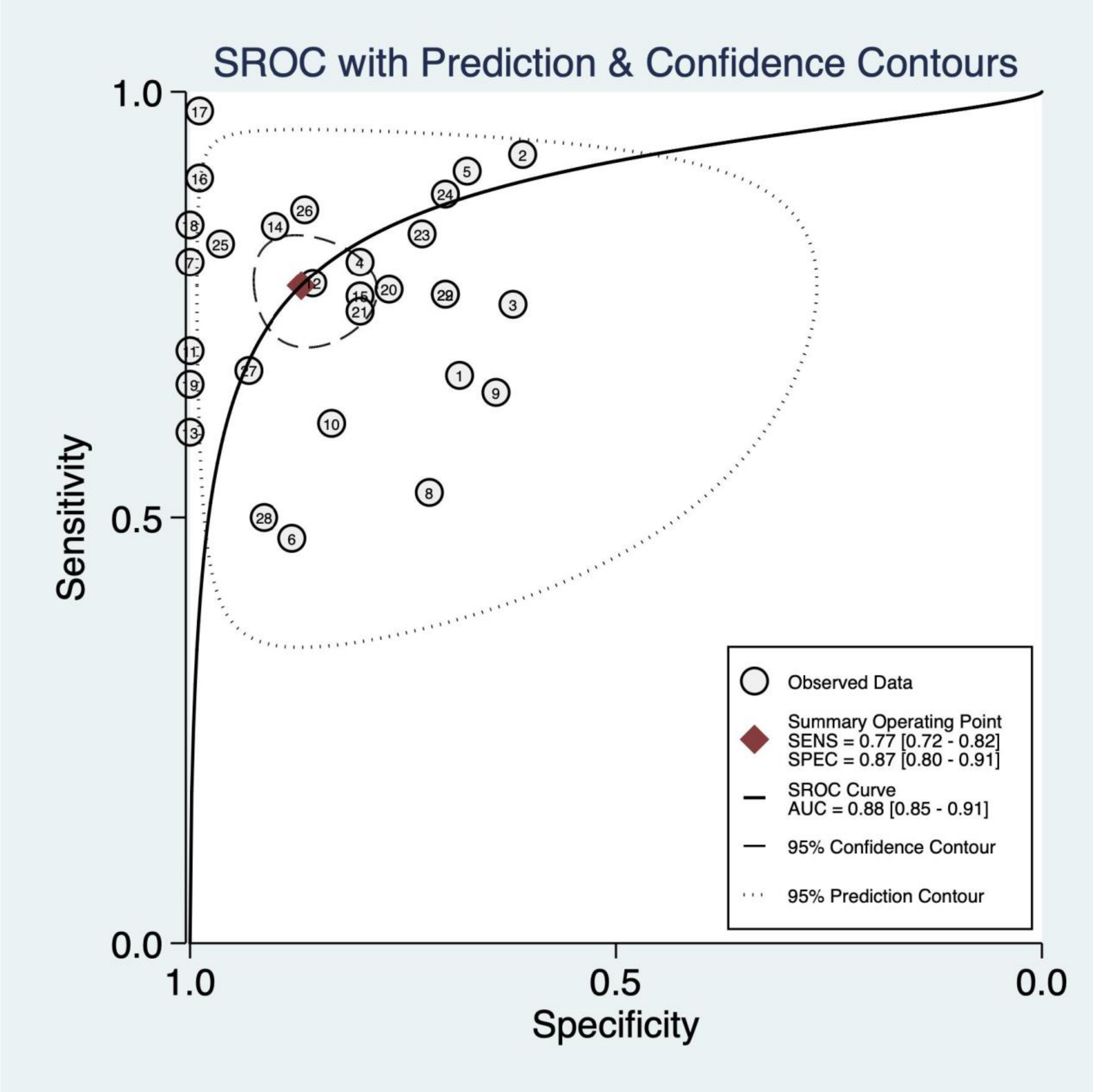

Figure S24. SROC curves of miRNA-21 for diagnosis of lung cancer (NSCLC).

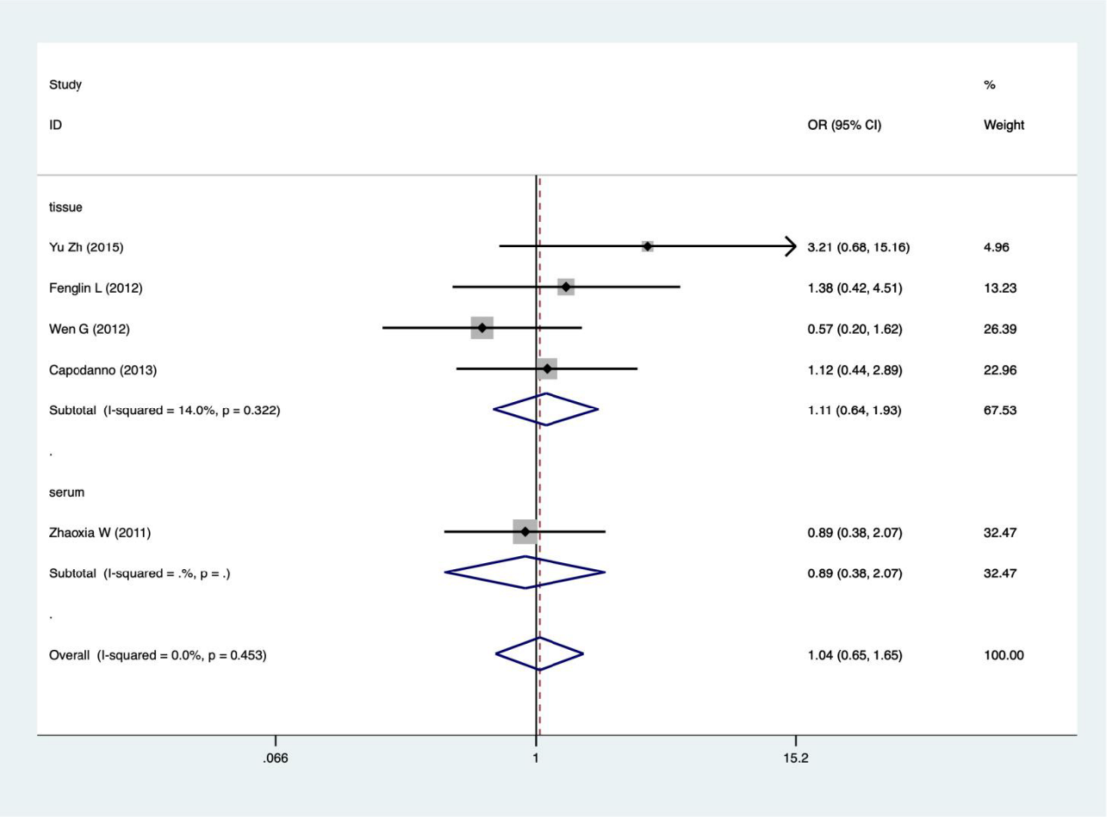

Figure S25. Forest plots of studies evaluating the correlation between miRNA-21 expression and lung cancer (Gender- subgroup of Specimen).

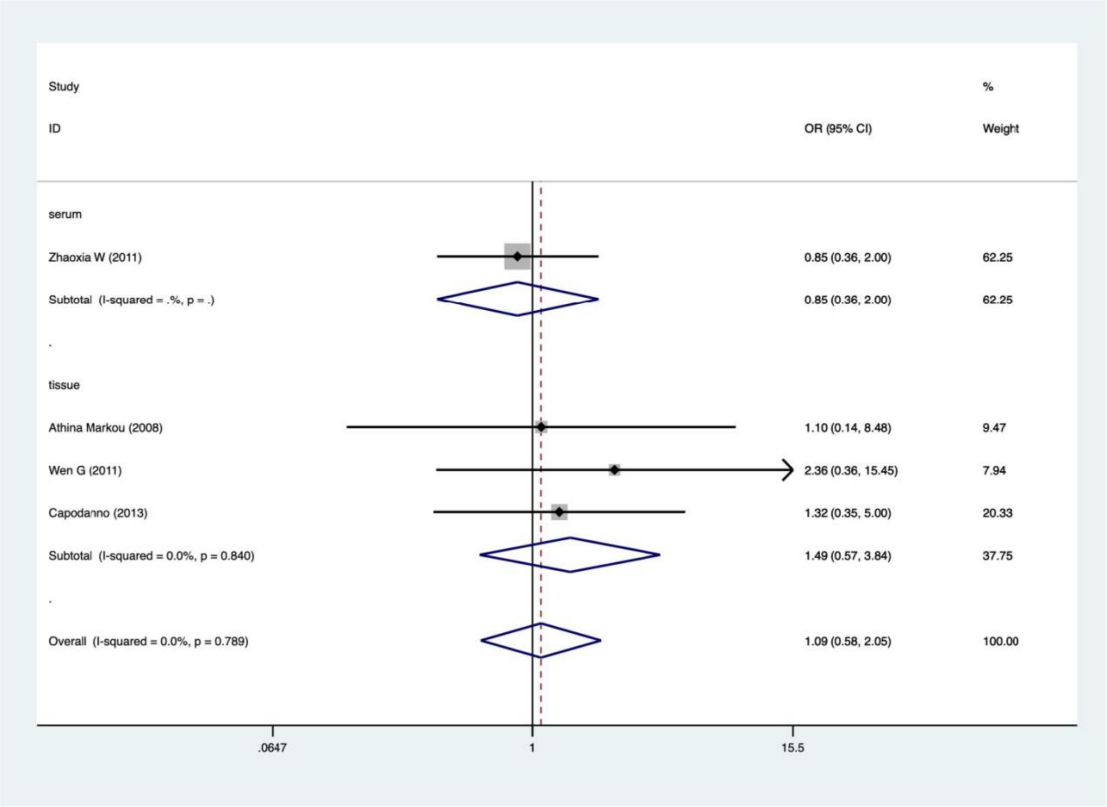

Figure S26. Forest plots of studies evaluating the correlation between miRNA-21 expression and lung cancer (Smoking History - subgroup of Specimen).

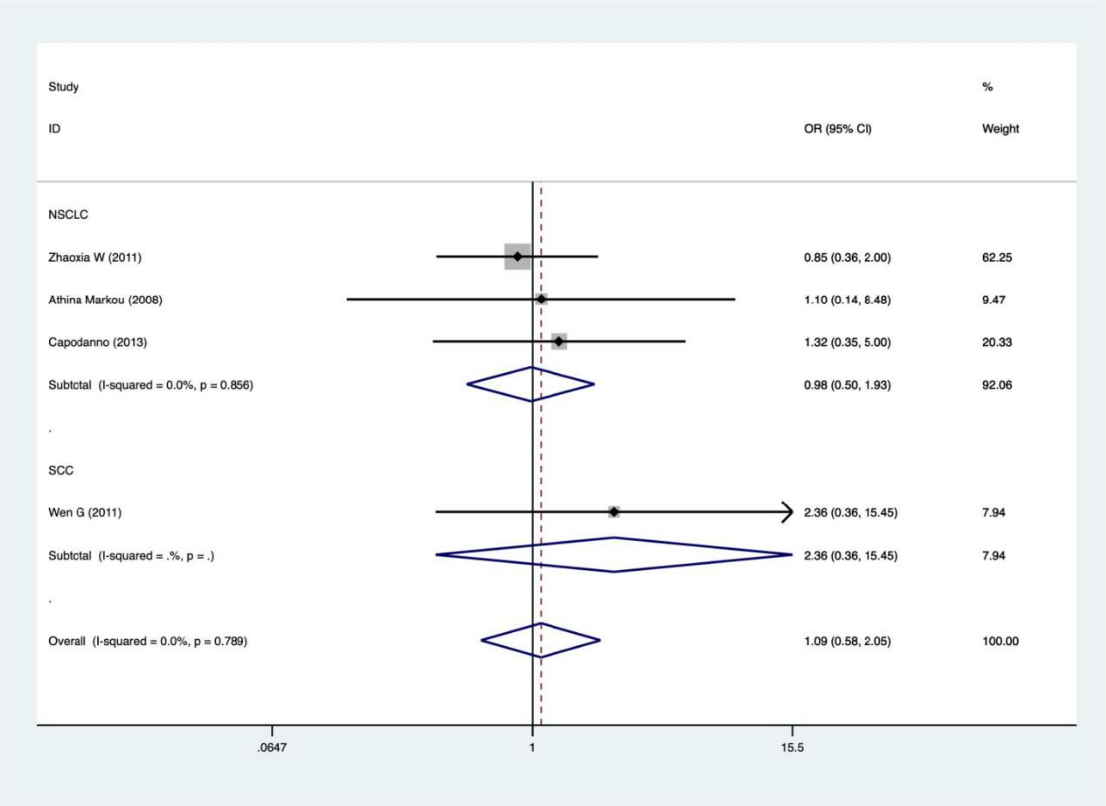

Figure S27. Forest plots of studies evaluating the correlation between miRNA-21 expression and lung cancer (Smoking History - subgroup of Cancer type).

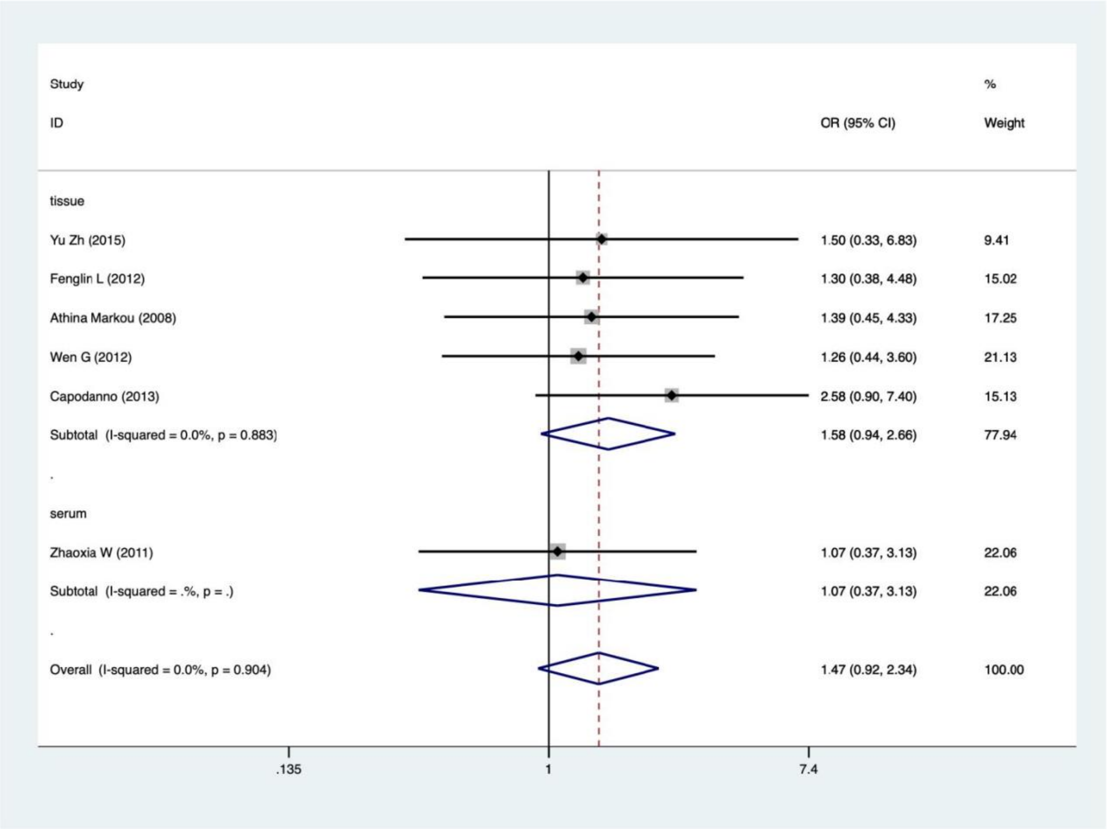

Figure S28. Forest plots of studies evaluating the correlation between miRNA-21 expression and lung cancer (Histological type - NSCLC).

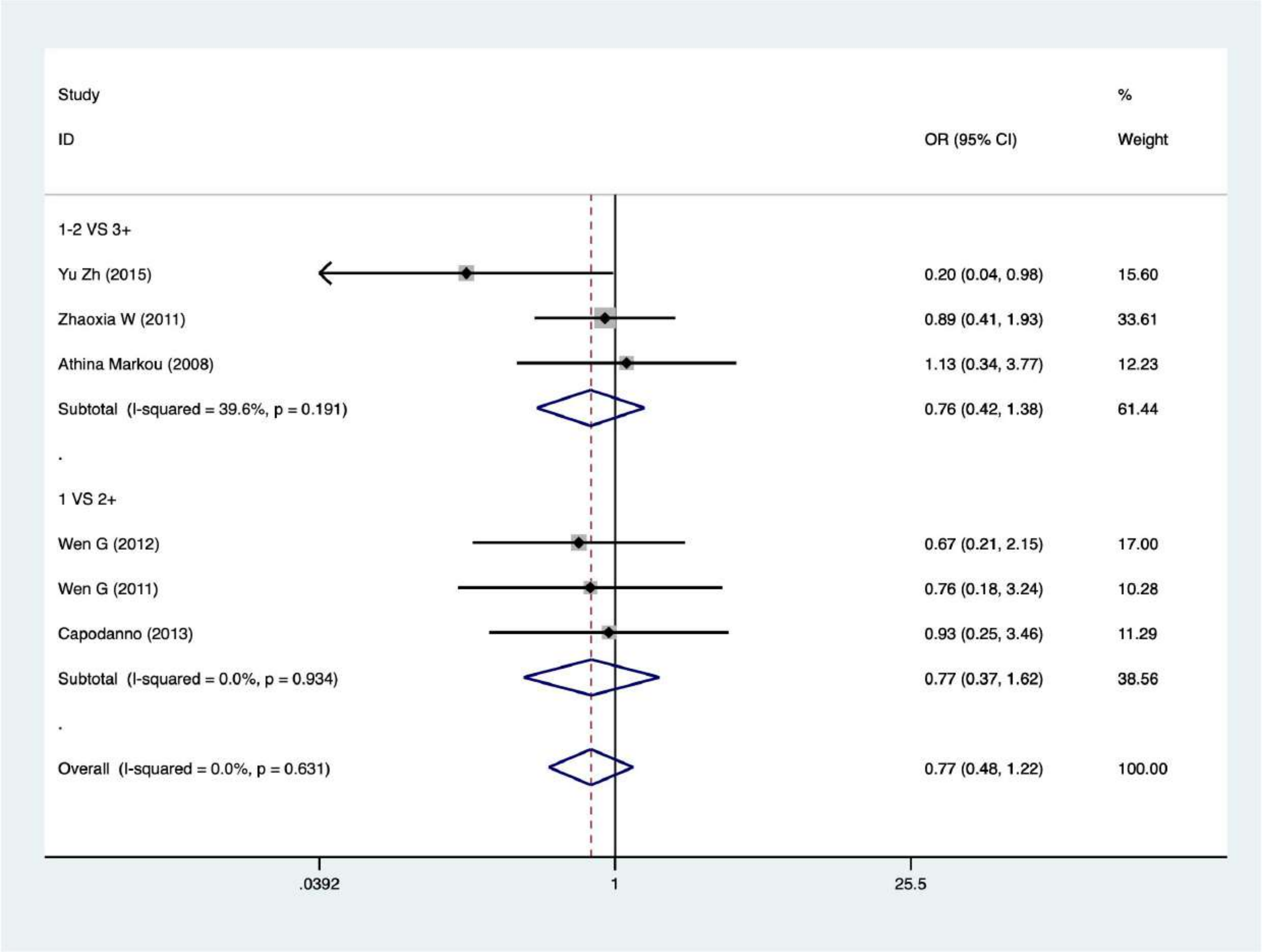

Study
ID
OR (95% CI)
%
Weight
1-2 VS 3+
Yu Zh (2015)
0.20 (0.04, 0.98)
15.60
Zhaoxia W (2011)
0.89 (0.41, 1.93)
33.61
Athina Markou (2008)
1.13 (0.34, 3.77)
12.23
Subtotal (I-squared = 39.6%, p = 0.191)
0.76 (0.42, 1.38)
61.44
.
1 VS 2+
Wen G (2012)
0.67 (0.21, 2.15)
17.00
Wen G (2011)
0.76 (0.18, 3.24)
10.28
Capodanno (2013)
0.93 (0.25, 3.46)
11.29
Subtotal (I-squared = 0.0%, p = 0.934)
0.77 (0.37, 1.62)
38.56
.
Overall (I-squared = 0.0%, p = 0.631)
0.77 (0.48, 1.22)
100.00
.0392
1
25.5

Figure S29. Forest plots of studies evaluating the correlation between the miRNA-21 expression and lung cancer (Pathological staging).

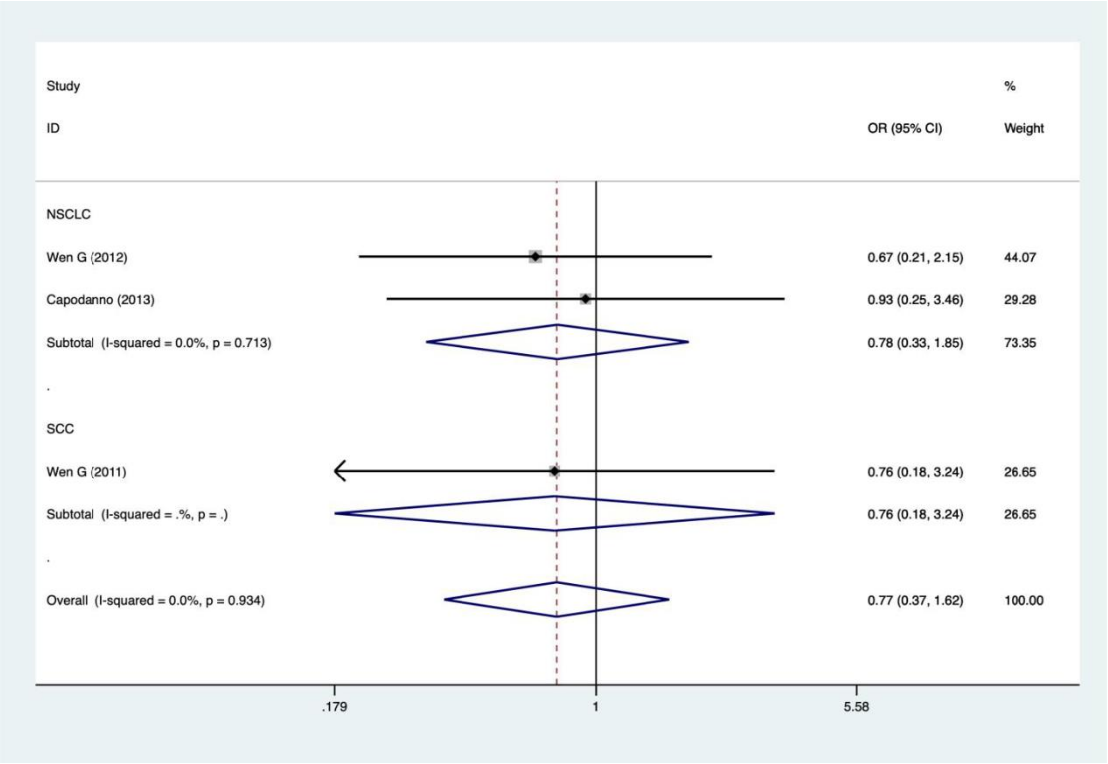

Figure S30. Forest plots of studies evaluating the correlation between miRNA-21 expression and lung cancer (Pathological staging - subgroup of “1 vs 2+”).

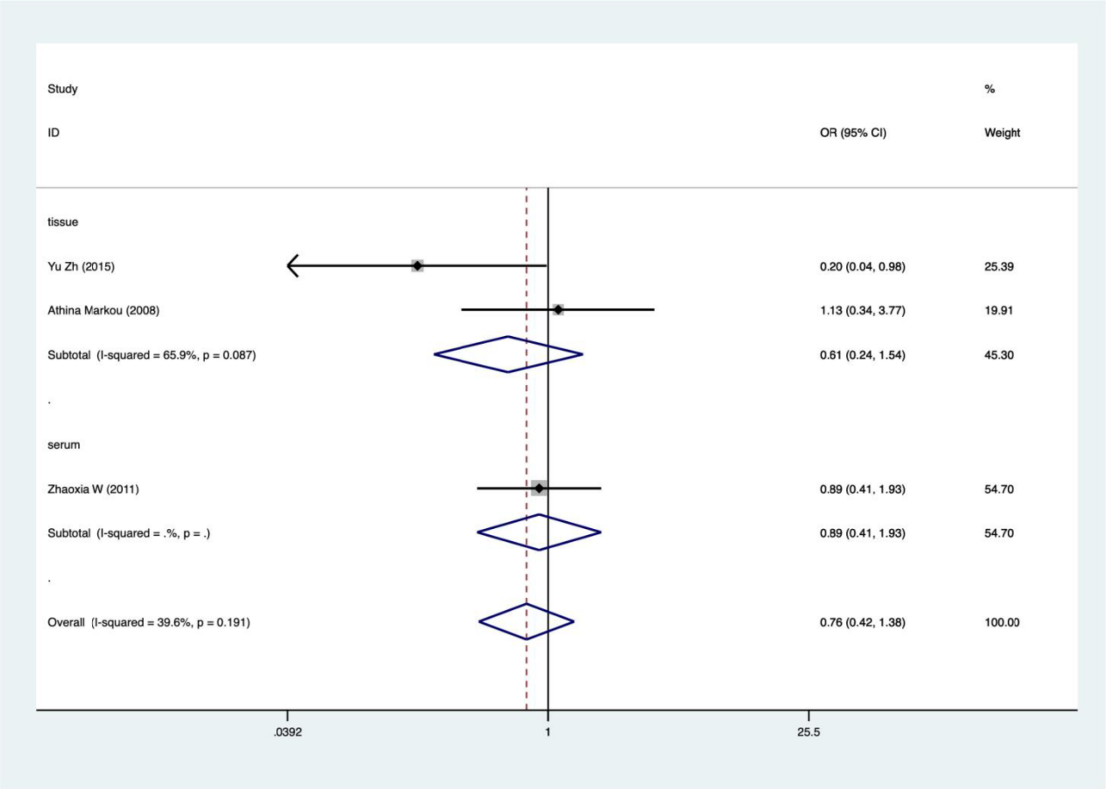

Figure S31. Forest plots of studies evaluating the correlation between miRNA-21 expression and lung cancer (Pathological staging - subgroup of “1-2 vs 3+”).

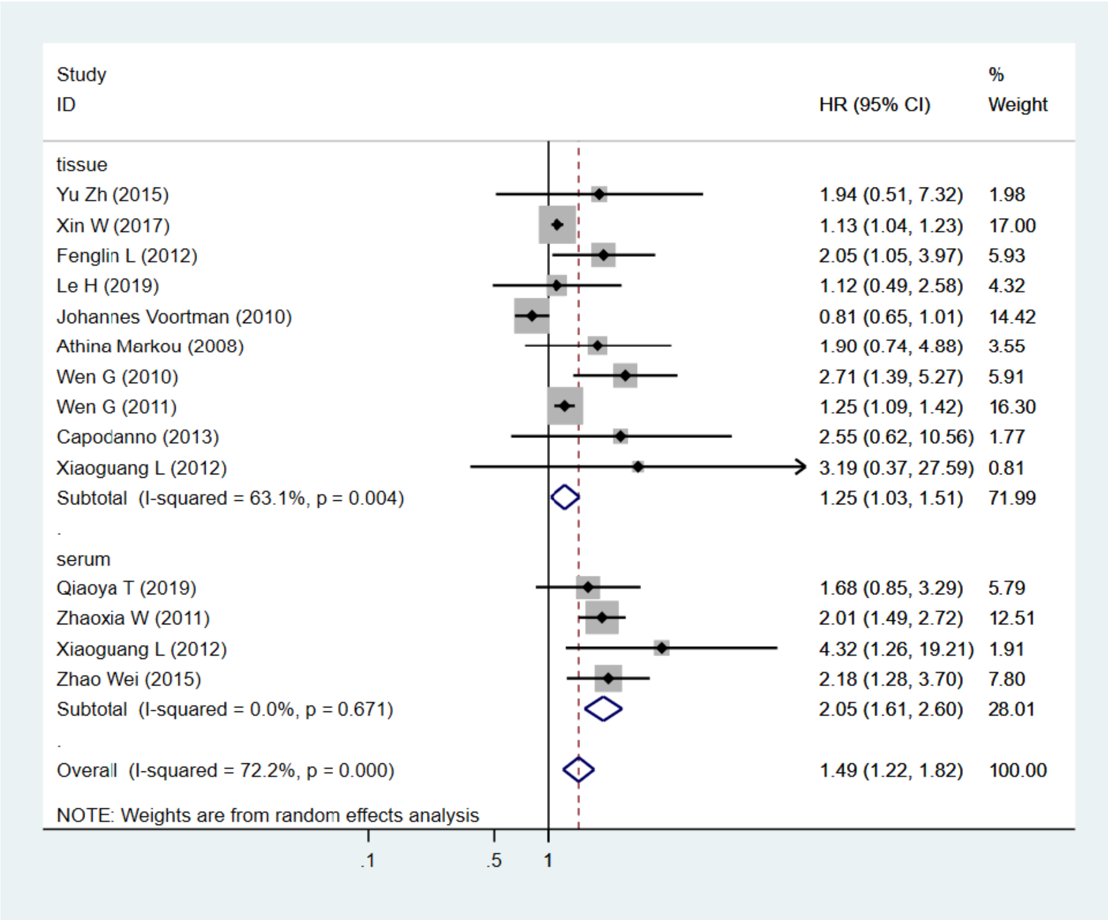

Figure S32. Forest plots on the role of miRNA-21 in prognosis of lung cancer (Univariate analysis of OS - subgroup of Specimen).

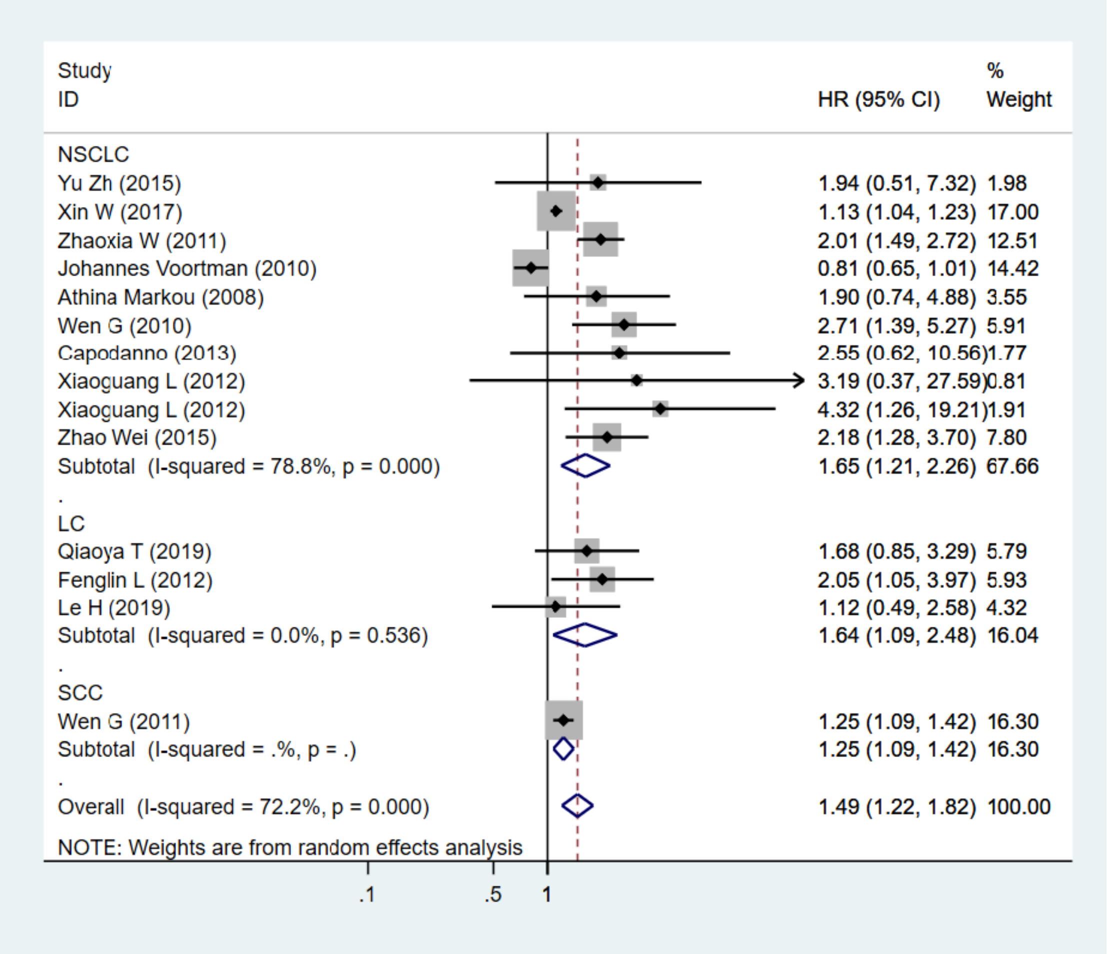

Figure S33. Forest plots on the role of miRNA-21 in prognosis of lung cancer (Univariate analysis of OS - subgroup of Cancer type).

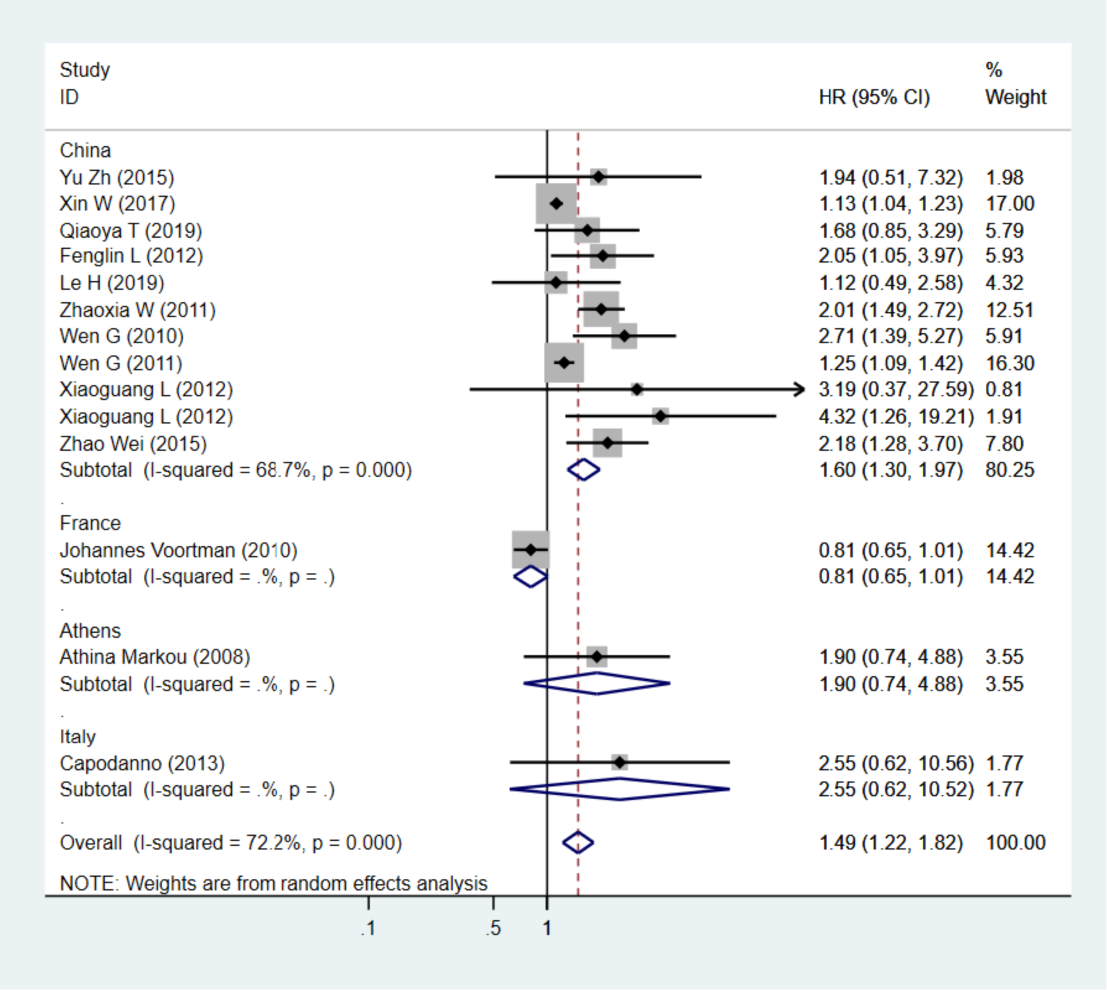

Figure S34. Forest plots on the role of miRNA-21 in prognosis of lung cancer (Univariate analysis of OS - subgroup of Country).

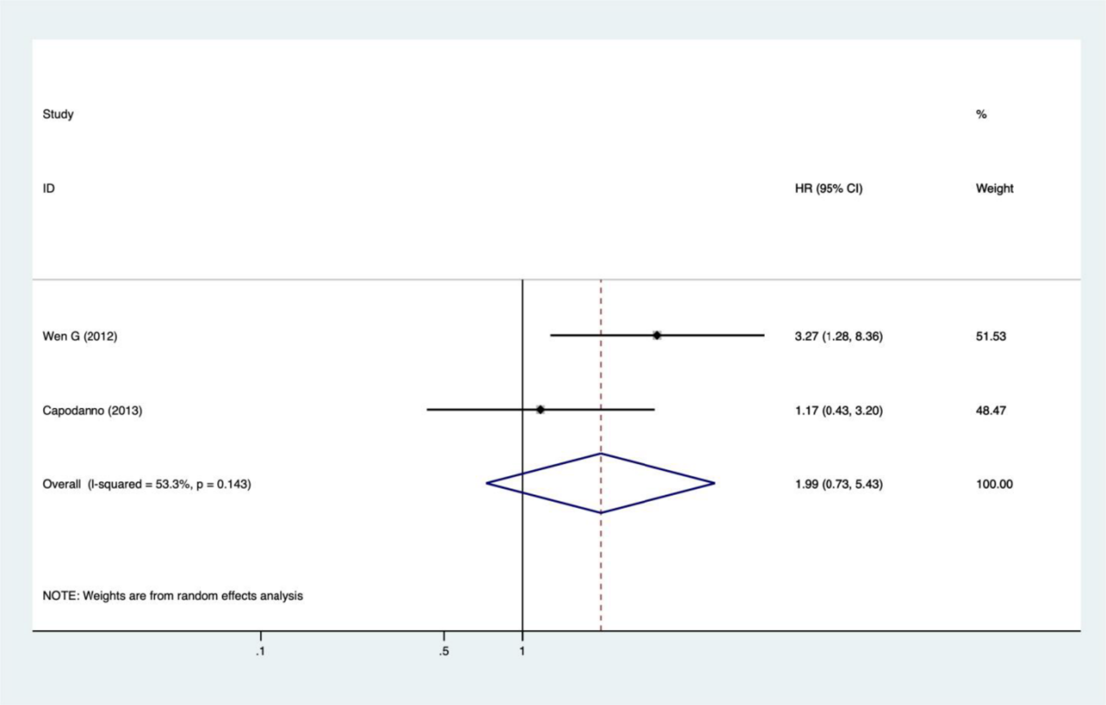

Figure S35. Forest plots on the role of miRNA-21 in prognosis of lung cancer (Univariate analysis of PFS/DFS).

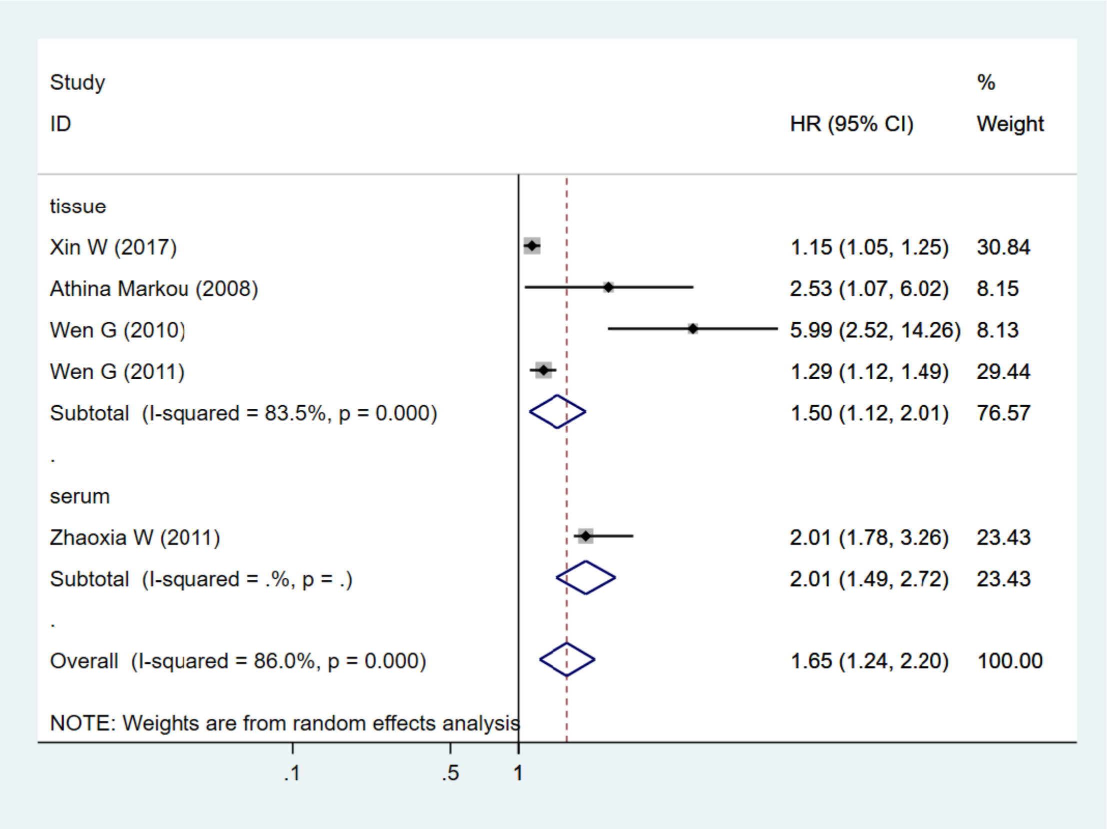

Figure S36. Forest plots on the role of miRNA-21 in prognosis of lung cancer (Multivariate analysis of OS - subgroup of Specimen).

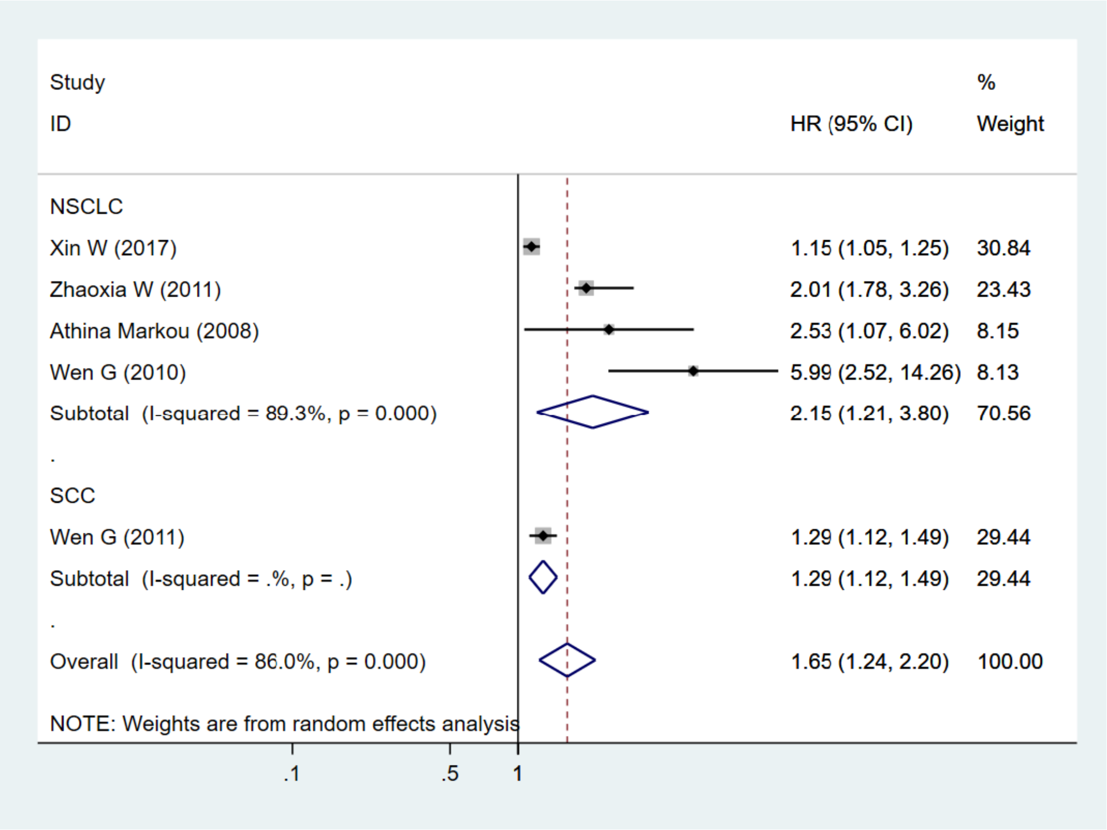

Figure S37. Forest plots on the role of miRNA-21 in prognosis of lung cancer (Multivariate analysis of OS - subgroup of Cancer type).

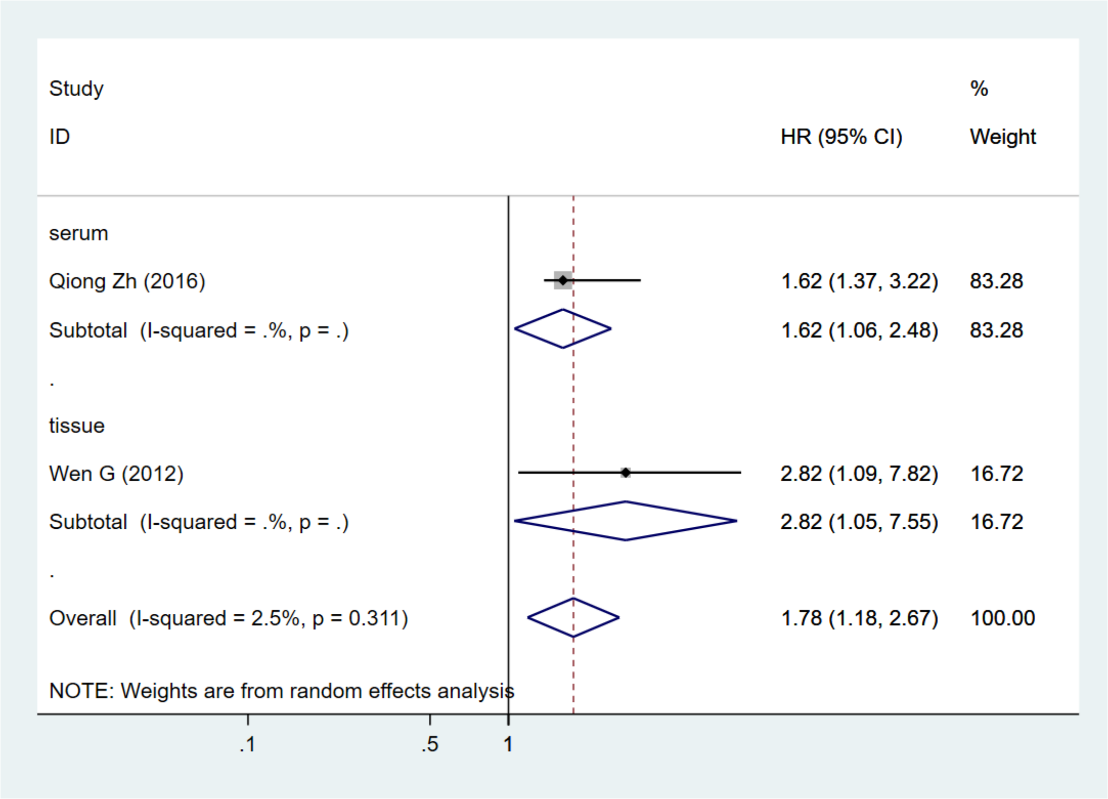

Figure S38. Forest plots on the role of miRNA-21 in prognosis of lung cancer (Multivariate analysis of PFS/DFS - subgroup of Specimen type).
